# Supplementary material for: Genes associated with ant social behavior show distinct transcriptional and evolutionary patterns
Source: eLife. 2015 Jan 26;4:e04775. doi: 10.7554/eLife.04775 (PMC4383337; doi:10.7554/eLife.04775)
Supplement: Supplementary file 2. — A PDF of the full R script and abbreviated output with the analyses included in the study. DOI: http://dx.doi.org/10.7554/eLife.04775.011 [file elife04775s002.pdf]

Complete analysis for Mikheyev and Linksvayer, Genes associated with ant social behavior show distinct transcriptional and evolutionary patterns

==

```r
library(ggplot2)
library(RMySQL)
library(edgeR)
library(DESeq2)
library(GOstats)
library(GSEABase)
library(gplots)
library(gmodels)
library(gtools)
library(pgirmess)
library(class)
library(gridExtra)
library(reshape)
library(WGCNA)
```

```
## ==========================================================================
## *
## *  Package WGCNA 1.41.1 loaded.
## *
## *    Important note: It appears that your system supports multi-threading,
## *    but it is not enabled within WGCNA in R.
## *    To allow multi-threading within WGCNA with all available cores, use
## *
## *          allowWGCNAThreads()
## *
## *    within R. Use disableWGCNAThreads() to disable threading if necessary.
## *    Alternatively, set the following environment variable on your system:
## *
## *          ALLOW_WGCNA_THREADS=<number_of_processors>
## *
## *    for example
## *
## *          ALLOW_WGCNA_THREADS=8
## *
## *    To set the environment variable in linux bash shell, type
## *
## *           export ALLOW_WGCNA_THREADS=8
## *
## *     before running R. Other operating systems or shells will
## *     have a similar command to achieve the same aim.
## *
## ==========================================================================
```

```r
allowWGCNAThreads()
```

```
## Allowing multi-threading with up to 8 threads.
```

```
library(boot)
library(multcomp)
library(data.table)
library(doMC)
registerDoMC(6)
```

Read gene expression data from SQL database and filter any gene with more than half of the libraries with FPKM<1.

```
if (exists("mydb")) dbDisconnect(mydb)
#mydb = dbConnect(MySQL(),  dbname='monomorium', host='ECOEVO.UNIT.OIST.JP')
mydb = dbConnect(MySQL(), user = "root",dbname = "monomorium1", host = "localhost")
contrasts <- dbGetQuery(mydb,"SELECT * FROM contrasts") # read a priori contrasts
# this makes the contrasts matrix compatible with design matrix, which is sorted by level
contrasts <- contrasts[,sort(names(contrasts))]
factors <- dbGetQuery(mydb,"SELECT * FROM factors")  #read treatments

#Note: MP12 (age15), MP14 (age18), MP19 (prot) have relatively low mapping rates (<70%)

counts <- dbGetQuery(mydb,"SELECT gene_id,MP1,MP2,MP3,MP4,MP5,MP6,MP7,MP8,MP9,MP10,MP11,MP12,MP13,
  MP14,MP15,MP16,MP17,MP18,MP19,MP20,MP21,MP22,MP23,MP24 FROM expected_counts_genes")
row.names(counts) <- counts$gene_id # change first column to row names
counts <- subset(counts,select=-c(gene_id))
fpkm <- dbGetQuery(mydb,"SELECT gene_id,MP1,MP2,MP3,MP4,MP5,MP6,MP7,MP8,MP9,MP10,MP11,MP12,MP13,
  MP14,MP15,MP16,MP17,MP18,MP19,MP20,MP21,MP22,MP23,MP24 FROM fpkm_genes")
row.names(fpkm) <- rownames(counts)
fpkm <- subset(fpkm,select=-c(gene_id))

#select isoforms where at least half of the libraries have FPKM >1
keep=rowSums(fpkm>= 1)>= ncol(counts)/2
#create design matrix, and label rows and columns according to our contrasts
design <- model.matrix(~0+factors$factor,data=counts)
rownames(design) <- colnames(counts)
colnames(design) <- names(contrasts)
```

Visualize cutoff distribution

```
ggplot(melt(fpkm),aes(x=value,color=variable))+geom_density()+
  scale_x_log10()+theme_bw()+geom_vline(xintercept=1, color="red")+
  xlab('expected counts')+ylab('density')+ theme(legend.position="none")
```

```
## Using  as id variables
```

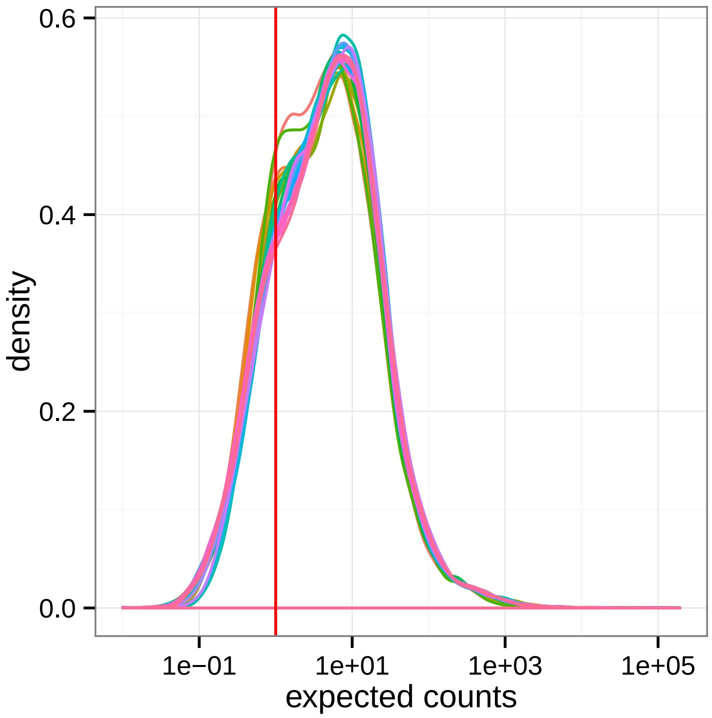

Conduct differential gene expression analysis

```
dge <- DGEList(counts=round(counts[keep,]),group=factors$factor)    #apply filtering!
dge <- calcNormFactors(dge)
dge <- estimateGLMCommonDisp(dge,design,verbose=TRUE)
```

```
## Disp = 0.02959 , BCV = 0.172
```

```
dge <- estimateGLMTrendedDisp(dge, design)
dge <- estimateGLMTagwiseDisp(dge, design)
fit <- glmFit(dge,design)
lrt <- glmLRT(fit)
```

plot NMDS as a sanity check

```
mdsplot <- plotMDS(dge,top=500)
```

```
plot(mdsplot, main="",xlab="Dimension 1",ylab="Dimension 2")
text(mdsplot$cmdscale.out[,1],mdsplot$cmdscale.out[,2],factors$factor)
```

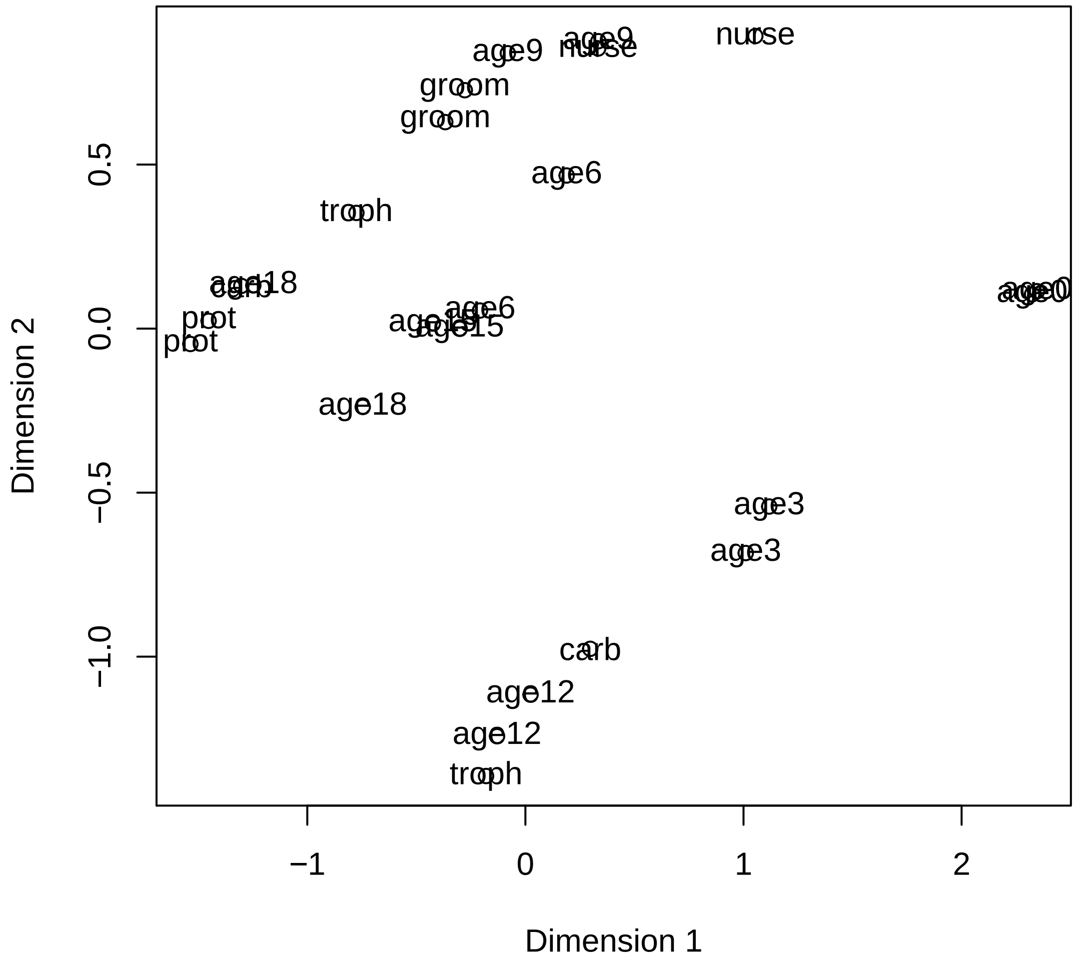

The ages and tasks cluster together, suggesting that the transcriptional data capture some sort of biological signal.

Examine task-specific gene expression, and determine which behaviors we should focus on

```r
#logFC is computed as X - Y, so positive values are upregulated in focal contast
et_forager <- glmLRT(fit, contrast=makeContrasts((carb+prot)/2 - (nurse+groom+troph)/3,
  levels=design))

par(mfrow=c(2,2))
summary(de_forager <- decideTestsDGE(et_forager, p=0.05, adjust="BH"))
```

```
##    [,1]
## -1   403
## 0  16380
## 1    588
```

```r
plotSmear(et_forager, de.tags=rownames(fit)[as.logical(de_forager)])
title("foragers vs. others")
et_nurse <- glmLRT(fit, contrast=makeContrasts(nurse - (carb+prot+groom+troph)/4, levels=design))
summary(de_nurse <- decideTestsDGE(et_nurse, p=0.05, adjust="BH"))
```

```
##    [,1]
## -1   898
## 0  15444
## 1   1029
```

```r
plotSmear(et_nurse, de.tags=rownames(fit)[as.logical(de_nurse)])
title("nurses vs. others")
et_groom <- glmLRT(fit, contrast=makeContrasts(groom - (carb+prot+nurse+troph)/4, levels=design))
summary(de_groom <- decideTestsDGE(et_groom, p=0.05, adjust="BH"))
```

```
##    [,1]
## -1     2
## 0  17343
## 1     26
```

```r
plotSmear(et_groom, de.tags=rownames(fit)[as.logical(de_groom)])
title("grooming vs. others")
et_troph <- glmLRT(fit, contrast=makeContrasts(troph - (carb+prot+nurse+groom)/4, levels=design))
summary(de_troph <- decideTestsDGE(et_troph, p=0.05, adjust="BH"))
```

```
##    [,1]
## -1    25
## 0  17323
## 1     23
```

```r
plotSmear(et_troph, de.tags=rownames(fit)[as.logical(de_troph)])
title("trophallaxis vs. others")
```

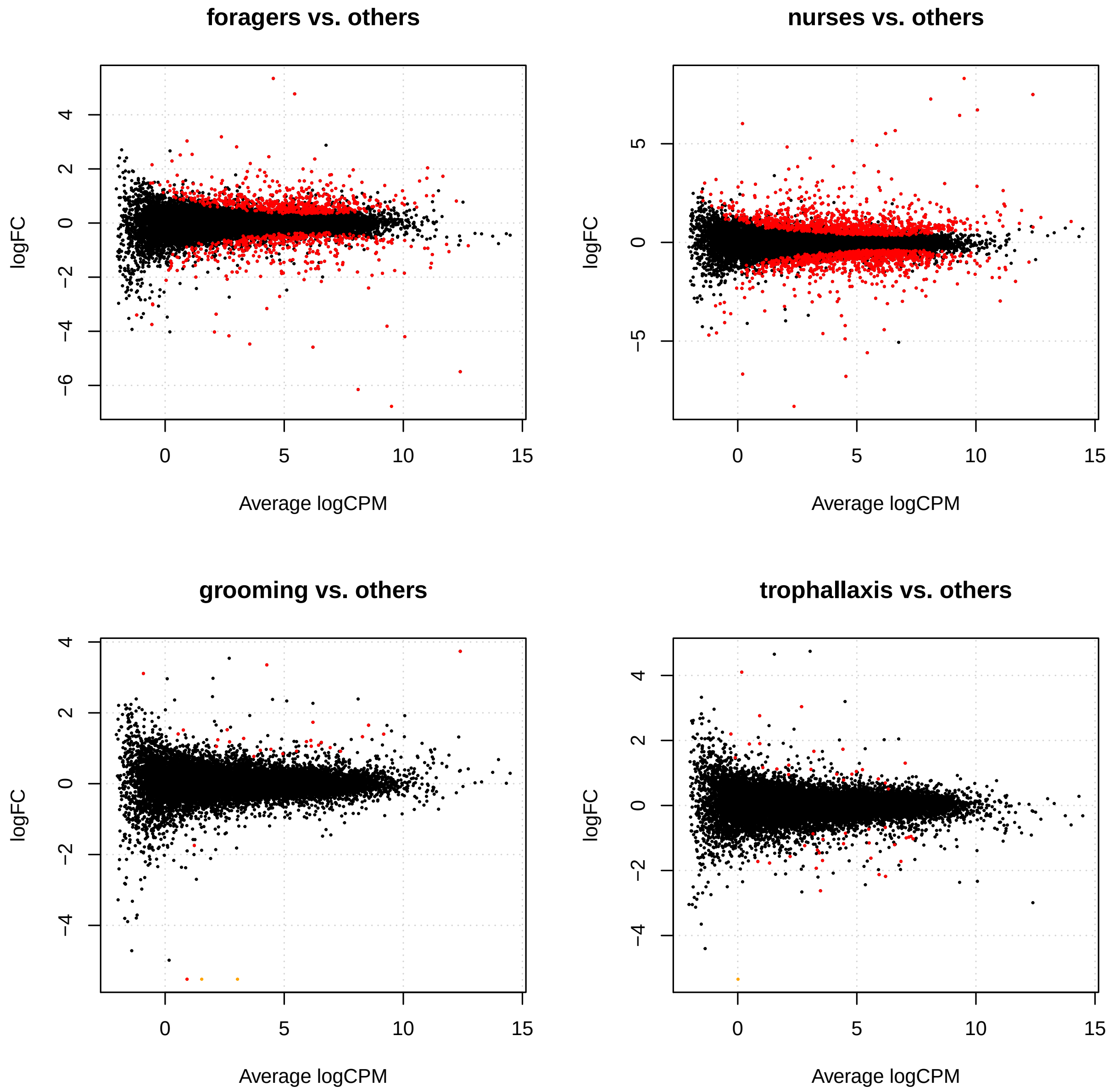


```r
par(mfrow=c(1,1))

et_nurse_forager <- glmLRT(fit, contrast=makeContrasts(nurse - (carb + prot)/2, levels=design))
summary(de_nurse_forager <- decideTestsDGE(et_nurse_forager, p=0.05, adjust="BH"))
```

```
##    [,1]
## -1  1217
## 0  14907
## 1   1247
```

Nurses and foragers are the two most distinct behavioral categories, and we'll focus our analysis on the from now on.

Comparison with fire ants

Test to see if evolutionary rates differ between forager-upregulated and nurse-upregulated genes.

```
dnds <- dbGetQuery(mydb,"SELECT * FROM dNdS") # read evolutionary rates vs S. invicta
nurse_names <- rownames(et_nurse_forager$table[de_nurse_forager == 1,])
forager_names <- rownames(et_nurse_forager$table[de_nurse_forager == -1,])
nde_names <- rownames(et_nurse_forager$table[de_nurse_forager == 0 ,])
rates <- data.frame(rate = c(dnds[na.omit(match(nurse_names,dnds$gene)),2],
    dnds[na.omit(match(forager_names,dnds$gene)),2],
    dnds[na.omit(match(nde_names,dnds$gene)),2]),
    task = c(rep("nurse",length(na.omit(match(nurse_names,dnds$gene)))),
        rep("forager",length(na.omit(match(forager_names,dnds$gene)))),
        rep("NDE",length(na.omit(match(nde_names,dnds$gene))))))
rates$task <- factor(rates$task, levels=c("NDE" ,"nurse","forager"))
ggplot(data=rates, aes(x=factor(task),y=rate))+geom_boxplot(notch=TRUE)+scale_y_log10()+theme_bw()
```

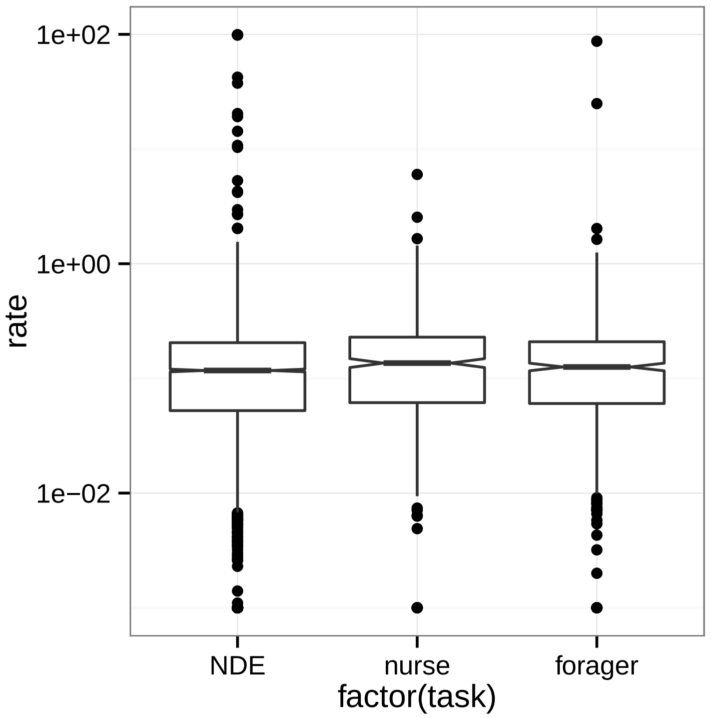


```
kruskal.test(rate ~ task, data = rates)
```

```
##
##  Kruskal-Wallis rank sum test
##
## data:  rate by task
## Kruskal-Wallis chi-squared = 10.48, df = 2, p-value = 0.005297
```

```
kruskalmc(rate ~ task, data = rates)
```

```
## Multiple comparison test after Kruskal-Wallis
```

```
## p.value: 0.05
## Comparisons
##               obs.dif critical.dif difference
## NDE-nurse       283.9        235.4       TRUE
## NDE-forager     153.7        215.6      FALSE
## nurse-forager   130.2        305.4      FALSE
```

```
#excluding
kruskal.test(rate ~ task, data = rates[rates$task != "NDE",])
```

```
##
##  Kruskal-Wallis rank sum test
##
## data:  rate by task
## Kruskal-Wallis chi-squared = 1.087, df = 1, p-value = 0.297
```

Looking at the data this way, it looks like nurse genes evolve significantly faster than NDE genes.

Are more highly expressed genes evolving slower?

```
cor.test(rowMeans(fpkm[dnds$gene,]),dnds$rate, method="spearman")
```

```
##
##  Spearman's rank correlation rho
##
## data:  rowMeans(fpkm[dnds$gene, ]) and dnds$rate
## S = 1.058e+11, p-value < 2.2e-16
## alternative hypothesis: true rho is not equal to 0
## sample estimates:
##     rho
## -0.1174
```

```
ggplot(data.frame(fpkm=rowMeans(fpkm[dnds$gene,]),rate=dnds$rate),aes(fpkm,rate))+
  geom_point(alpha=0.2)+scale_x_log10()+scale_y_log10()+theme_bw()
```

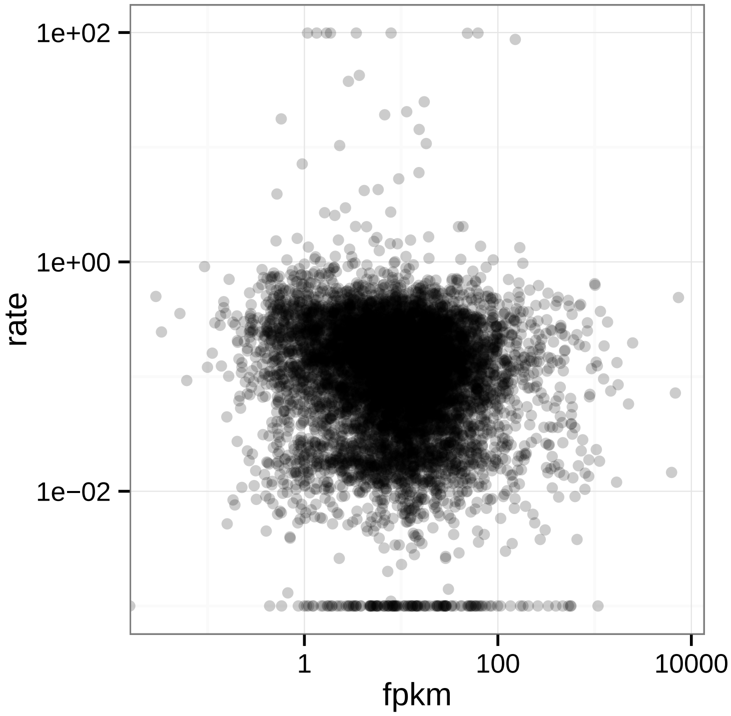

Yes, it looks like more highly expressed genes are indeed evolving slower.

Are forager genes or nurse, genes more highly expressed?

- *caveat*: more power to detect differential expression in highly expressed genes

```
fpkm_task <- data.frame(fpkm = rowMeans(fpkm[rownames(lrt$table),]), task = de_nurse_forager )
fpkm_task[forager_names,"task"] <- "forager"
fpkm_task[nurse_names,"task"] <- "nurse"
fpkm_task[fpkm_task[,"task"] == 0,"task"] <- "nde"
kruskalmc(fpkm ~ task, fpkm_task)
```

```
## Multiple comparison test after Kruskal-Wallis
## p.value: 0.05
## Comparisons
##                 obs.dif critical.dif difference
## forager-nde      2742.2        357.9       TRUE
## forager-nurse     745.7        483.7       TRUE
## nde-nurse        1996.5        353.9       TRUE
```

```
ggplot(fpkm_task,aes(x=factor(task),y=log(fpkm)))+geom_boxplot()+theme_bw()+
  xlab("")+ylab("log fragments per kilobase mapped (FPKM)")
```

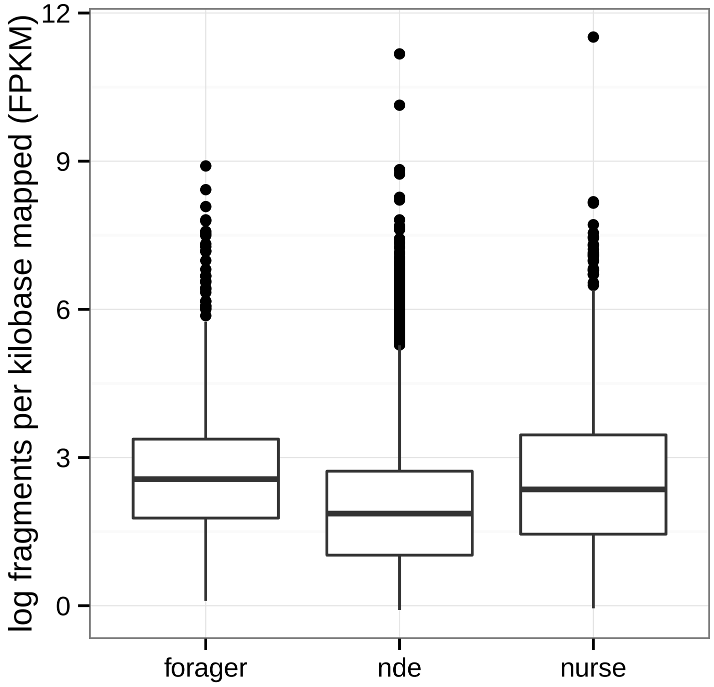

Comparing results with fire ant foraging study by Manfredini et al 2013

```
manfredini <- dbGetQuery(mydb, '
SELECT DISTINCT mp_id, microarray.gene_id, expression,rate FROM `mp vs sinv`
JOIN dNdS
ON `mp vs sinv`.mp_id = dNdS.gene_id
LEFT JOIN (SELECT DISTINCT gene_id, IF (logFC>0,"forager","nest") AS expression FROM
manfredini_expression
JOIN manfredini_names
ON manfredini_expression.manfredini_id = manfredini_names.manfredini_id) AS microarray
ON sinv_id = microarray.gene_id
GROUP BY mp_id
') #a few probes target the same gene, so just choose one of them
rownames(manfredini) <- manfredini$mp_id
fisher.test(matrix(c(
    nrow(subset(manfredini, expression=="forager")), # number of sinv forager genes with mp homologs
    nrow(manfredini[is.na(manfredini$expression),]),
    table(forager_names %in% subset(manfredini,expression=="forager")$mp_id)["TRUE"],
    table(forager_names %in% subset(manfredini,expression=="forager")$mp_id)["FALSE"]),
  ncol=2,byrow=T),alternative="less")
```

```
##
##  Fisher's Exact Test for Count Data
##
## data:
## p-value = 0.04031
## alternative hypothesis: true odds ratio is less than 1
## 95 percent confidence interval:
##  0.0000 0.9806
```

```
## sample estimates:
## odds ratio
##     0.7142
```

```r
fisher.test(matrix(c(
    nrow(subset(manfredini, expression=="nest")), # number of sinv nest genes with mp homologs
    nrow(manfredini[!is.na(manfredini$expression),]),
    table(nurse_names %in% subset(manfredini,expression=="nest")$mp_id)["TRUE"],
    table(nurse_names %in% subset(manfredini,expression=="nest")$mp_id)["FALSE"]),ncol=2,byrow=T),
  alternative="less")
```

```
##
##  Fisher's Exact Test for Count Data
##
## data:
## p-value = 1
## alternative hypothesis: true odds ratio is less than 1
## 95 percent confidence interval:
##   0.00 17.26
## sample estimates:
## odds ratio
##       12.7
```

There is a small, but significant overlap in the genes conserved in various contexts

Another way to look at this, is to look at the *direction* of genes, rather than their overall significance, or correlation between logFC

```r
manfredini2 <- dbGetQuery(mydb, '
SELECT DISTINCT mp_id, microarray.id, expression, logFC FROM `mp vs sinv`
JOIN dNdS ON `mp vs sinv`.mp_id = dNdS.gene_id
JOIN (SELECT manfredini_probe2gene.probe, manfredini_probe2gene.gene AS id,
IF(AVG(manfredini_expt1.out)>AVG(manfredini_expt1.in),"forager","nurse") AS expression,
log(2,POW(2,manfredini_expt1.in)/POW(2,manfredini_expt1.out)) as logFC
FROM  manfredini_expt1
  JOIN manfredini_probe2gene
    ON manfredini_expt1.probe = manfredini_probe2gene.probe GROUP BY id) AS microarray
ON sinv_id = microarray.id
')

rownames(manfredini2) <- manfredini2$mp_id
upreg <- et_nurse_forager$table
upreg$task <- "nurse"
upreg[upreg$logFC<0,"task"] <- "forager"
commonNames <- intersect(rownames(upreg),manfredini2$mp_id)
CrossTable(upreg[commonNames,"task"],manfredini2[commonNames,"expression"],fisher=T)
```

```
##
##
##    Cell Contents
## |-------------------------|
## |                       N |
## | Chi-square contribution |
```

```
## |           N / Row Total |
## |           N / Col Total |
## |         N / Table Total |
## |-------------------------|
##
##
## Total Observations in Table:  6324
##
##
##                            | manfredini2[commonNames, "expression"]
## upreg[commonNames, "task"] |   forager |     nurse | Row Total |
## ---------------------------|-----------|-----------|-----------|
##                    forager |      2137 |      1148 |      3285 |
##                            |    14.758 |    22.015 |           |
##                            |     0.651 |     0.349 |     0.519 |
##                            |     0.564 |     0.452 |           |
##                            |     0.338 |     0.182 |           |
## ---------------------------|-----------|-----------|-----------|
##                      nurse |      1649 |      1390 |      3039 |
##                            |    15.953 |    23.797 |           |
##                            |     0.543 |     0.457 |     0.481 |
##                            |     0.436 |     0.548 |           |
##                            |     0.261 |     0.220 |           |
## ---------------------------|-----------|-----------|-----------|
##               Column Total |      3786 |      2538 |      6324 |
##                            |     0.599 |     0.401 |           |
## ---------------------------|-----------|-----------|-----------|
##
##
## Fisher's Exact Test for Count Data
## ------------------------------------------------------------
## Sample estimate odds ratio:  1.569
##
## Alternative hypothesis: true odds ratio is not equal to 1
## p =  2.36e-18
## 95% confidence interval:  1.416 1.738
##
## Alternative hypothesis: true odds ratio is less than 1
## p =  1
## 95% confidence interval:  0 1.71
##
## Alternative hypothesis: true odds ratio is greater than 1
## p =  1.305e-18
## 95% confidence interval:  1.439 Inf
##
##
##
```

```
cor.test(upreg[commonNames,"logFC"],manfredini2[commonNames,"logFC"],method="spearman")
```

```
##
##  Spearman's rank correlation rho
##
## data:  upreg[commonNames, "logFC"] and manfredini2[commonNames, "logFC"]
```

```
## S = 3.612e+10, p-value < 2.2e-16
## alternative hypothesis: true rho is not equal to 0
## sample estimates:
##    rho
## 0.1432
```

So, about 55.8% of the genes are expressed in concordant direction and the correlation in logFC between the two datasets is rho=0.143

Comparing patterns of gene expression with honeybees

```
hbee <- dbGetQuery(mydb, '
SELECT `blast amel vs mp`.amel, `blast amel vs mp`.mp,`alaux dge`.task FROM
`blast amel vs mp` JOIN `blast mp vs amel`
ON `blast amel vs mp`.amel = `blast mp vs amel`.amel AND `blast amel vs mp`.mp =
`blast mp vs amel`.mp
LEFT JOIN `alaux dge`
ON `alaux dge`.gene = SUBSTR(`blast amel vs mp`.amel,1,7)')

# test to see whether there is an enrichment in mp forager genes that have bee homologs
fisher.test(matrix(c(
    nrow(subset(hbee, task=="forager")), # number of honeybee forager genes with mp homologs
    nrow(hbee[is.na(hbee$task),]),
    table(forager_names %in% subset(hbee,task=="forager")$mp)["TRUE"],
    table(forager_names %in% subset(hbee,task=="forager")$mp)["FALSE"]),ncol=2,byrow=T),
  alternative="less")
```

```
##
##  Fisher's Exact Test for Count Data
##
## data:
## p-value = 0.9775
## alternative hypothesis: true odds ratio is less than 1
## 95 percent confidence interval:
##  0.000 1.794
## sample estimates:
## odds ratio
##      1.352
```

```
fisher.test(matrix(c(
    nrow(subset(hbee, task=="nurse")),  # total hb nurse gene count
    nrow(hbee[is.na(hbee$task),]),      # number of NDE
    table(nurse_names %in% subset(hbee,task=="nurse")$mp)["TRUE"],
  # nurse genes matching mp nurse genes
    table(nurse_names %in% subset(hbee,task=="nurse")$mp)["FALSE"]),ncol=2,byrow=T),
  alternative="less") # genes not matching mp nurse genes
```

```
##
##  Fisher's Exact Test for Count Data
##
## data:
```

```
## p-value = 0.9997
## alternative hypothesis: true odds ratio is less than 1
## 95 percent confidence interval:
##  0.000 1.901
## sample estimates:
## odds ratio
##      1.517
```

By contrast with fire ants, there is no significant overlap with bees.

Plot heatmap for foragers, nurse and ages

- This heatmap shows just the top genes, so that the patterns are easier to see by eye

```
polyethism <- grepl("age|nurse|forager",gsub("carb|prot","forager",factors$factor))
forager_nurse_et_names <- rownames(et_nurse_forager$table[de_nurse_forager !=0,])
#plot the 20 genes most differentiated between nurses and workers
strong_dge <- order(et_nurse_forager$table[forager_nurse_et_names,"PValue"],decreasing=FALSE)[0:200]
strong_names <- rownames(et_nurse_forager$table[forager_nurse_et_names,][strong_dge,])
col_order <- mixedorder(gsub("nurse","0",factors$factor[polyethism]))
colData <- data.frame(task = factors$factor[polyethism][col_order],
    lib = colnames(counts)[polyethism][col_order])
```

```
mycols <- colorpanel(n=19,low="white",high="darkgreen")
color_codes <- data.frame(factor = c("age0", "age3", "age6", "age9", "age12", "age15", "age18",
"groom", "nurse", "troph", "prot","carb"), color = c("#CCFFFF","#66CCFF","#3399FF",
"#0066FF","#0000FF","#0000CC","#000066","#d53e4f","#FFFF00","#fee08b","#660066","#660066"))
colors <-  as.vector(color_codes$color[match(factors$factor[polyethism][col_order],
    color_codes$factor)])
heatmap.2(log(1+as.matrix(counts[strong_names,polyethism][1:20,col_order])),key=FALSE,
trace="none",col=mycols,labRow=FALSE,density.info="none",Colv=FALSE,
ColSideColors=as.vector(colors),labCol=factors$factor[polyethism][col_order],
hclustfun=function(c){hclust(c, method="ward")},margins = c(10, 2))
```

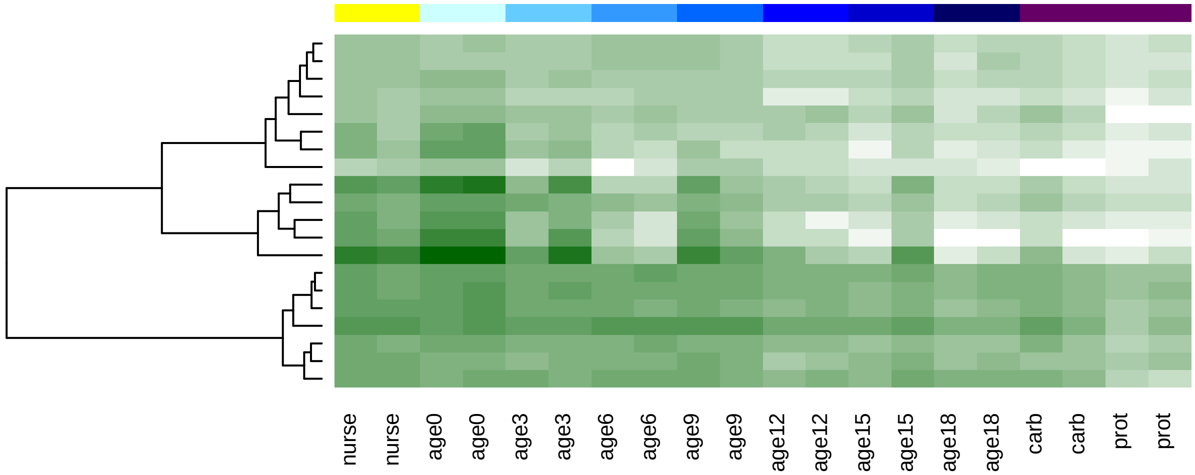


```
#plot the top 100 genes most differentiated, with an added dendrogram
#showing the relationships among all samples based on these 100 genes
heatmap.2(log(1+as.matrix(counts[strong_names,polyethism][1:100,col_order])),
key=FALSE,trace="none",col=mycols,labRow=FALSE,density.info="none",
ColSideColors=as.vector(colors),labCol=factors$factor[polyethism][col_order],
hclustfun=function(c){hclust(c, method="complete")},margins = c(10, 2))
```

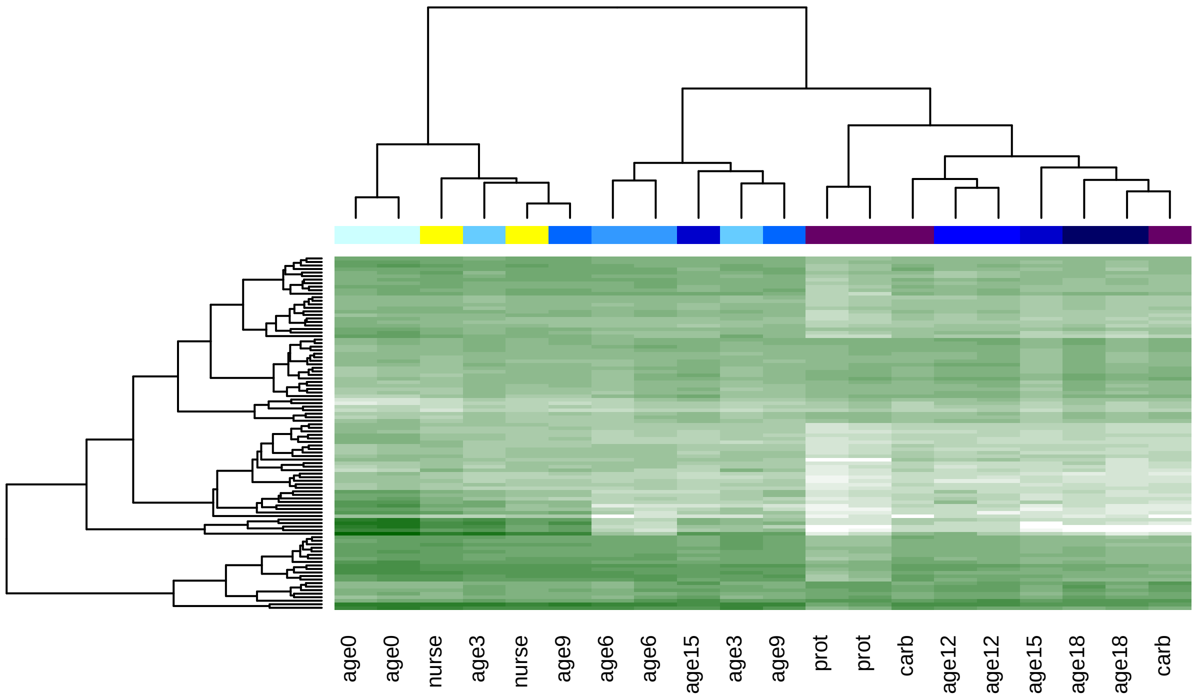

K-NN

- supervised learning of task-specific genes.

```
forager_nurse <- grepl("nurse|carb|prot",factors$factor)
tasks <- ! grepl("age",factors$factor)
train <- as.data.frame(t(counts[forager_nurse_et_names,forager_nurse]))
test <- as.data.frame(t(counts[forager_nurse_et_names,! tasks]))
cl <- factor(gsub("carb|prot","forager",factors$factor[forager_nurse]))
model <- knn(train, test, cl, k = 3, prob=TRUE)
cbind(model,factors$factor[! tasks],attr(model,"prob"))[mixedorder(factors$factor[! tasks]),]
```

```
##       model
##  [1,] "2"   "age0"  "0.666666666666667"
##  [2,] "2"   "age0"  "0.666666666666667"
##  [3,] "2"   "age3"  "0.666666666666667"
##  [4,] "2"   "age3"  "0.666666666666667"
##  [5,] "2"   "age6"  "0.666666666666667"
##  [6,] "2"   "age6"  "0.666666666666667"
##  [7,] "2"   "age9"  "0.666666666666667"
##  [8,] "2"   "age9"  "0.666666666666667"
##  [9,] "1"   "age12" "1"
## [10,] "1"   "age12" "1"
## [11,] "1"   "age15" "1"
```

```
## [12,] "1"   "age15" "0.666666666666667"
## [13,] "1"   "age18" "1"
## [14,] "1"   "age18" "1"
```

```
#visually see if the ages actually cluster together
temp<-t(counts[forager_nurse_et_names,! tasks])
rownames(temp) <- factors[!tasks,"factor"]
plot(hclust((dist(temp))))
```

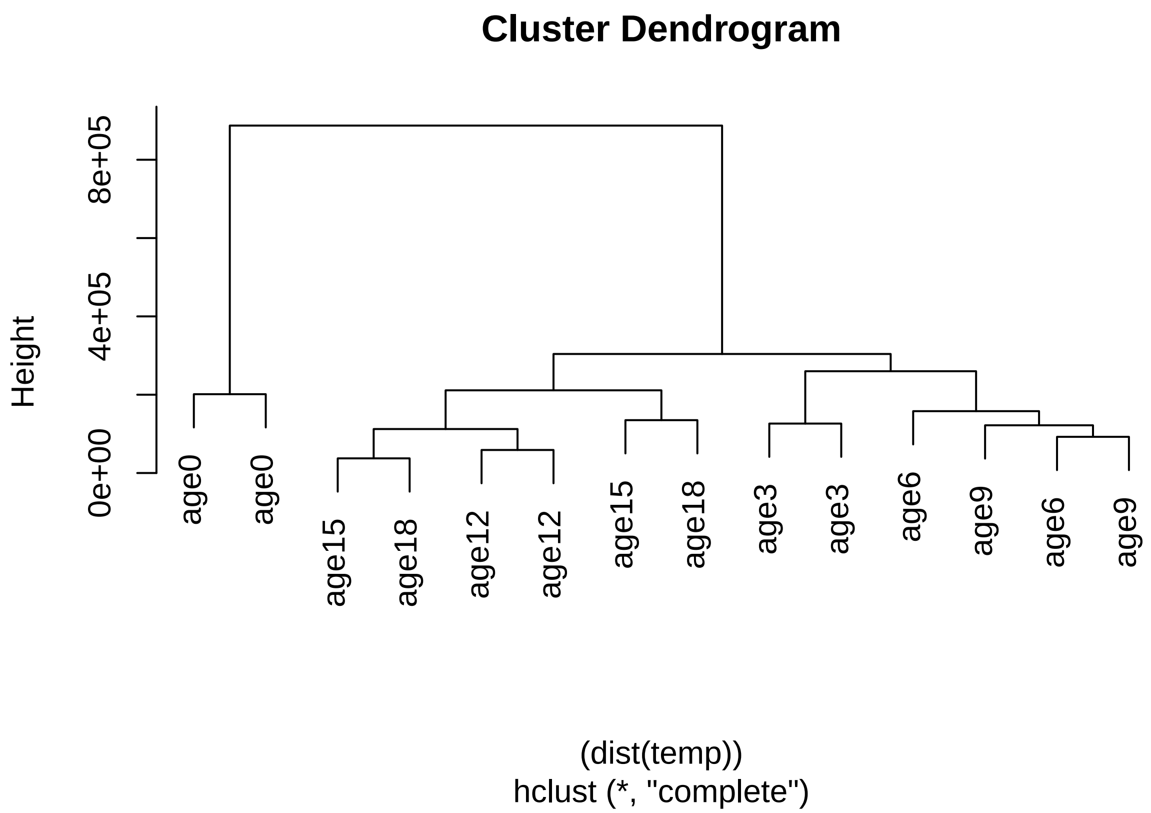


```
#excluding age0
model2 <- knn(train, test, cl, k = 3, prob=TRUE)
test2 <- as.data.frame(t(counts[forager_nurse_et_names,! tasks]))
model <- knn(train, test, cl, k = 3, prob=TRUE)
cbind(model2,factors$factor[! tasks],attr(model,"prob"))[mixedorder(factors$factor[! tasks]),]
```

```
##      model2
##  [1,] "2"    "age0"  "0.666666666666667"
##  [2,] "2"    "age0"  "0.666666666666667"
##  [3,] "2"    "age3"  "0.666666666666667"
##  [4,] "2"    "age3"  "0.666666666666667"
##  [5,] "2"    "age6"  "0.666666666666667"
##  [6,] "2"    "age6"  "0.666666666666667"
##  [7,] "2"    "age9"  "0.666666666666667"
##  [8,] "2"    "age9"  "0.666666666666667"
##  [9,] "1"    "age12" "1"
```

```
## [10,] "1"    "age12" "1"
## [11,] "1"    "age15" "1"
## [12,] "1"    "age15" "0.666666666666667"
## [13,] "1"    "age18" "1"
## [14,] "1"    "age18" "1"
```

k==2 and k==3 give close to the same result. See here for more details on knn and choosing k, and here for a justification of knn as an appropriate tool for classifying gene expression

Go term enrichment for forager- and nurse-upregulated genes, output to table

```r
#load go terms, and append
go <- dbGetQuery(mydb,'SELECT gene, GO FROM blast2go JOIN genes_isoforms ON
genes_isoforms.isoform = blast2go.isoform WHERE go != ""')
go$evidence <- "ISS"
go <- go[,c("GO","evidence","gene")]
dbDisconnect(mydb)
```

```
## [1] TRUE
```

```r
universe <- unique(go$gene)
goFrame=GOFrame(go[go$gene %in% universe,],organism="Monomorium pharaonis")
goAllFrame=GOAllFrame(goFrame)
gsc <- GeneSetCollection(goAllFrame, setType = GOCollection())

# GO terms overrepresented in forager-upregulated genes
forager_upreg_go <- hyperGTest(GSEAGOHyperGParams(name = "worker upregulated",
    geneSetCollection=gsc,geneIds = intersect(forager_names,universe),
    universeGeneIds=universe,ontology = "BP",pvalueCutoff = 0.05,
  conditional = FALSE,testDirection = "over"))
go_forager_up<-cbind(summary(forager_upreg_go),"forager_upregulated_genes","overrepresented")
colnames(go_forager_up)<-c("GOBPID","Pvalue","OddsRatio","ExpCount",
  "Count","Size","GOTerm","GeneCategory","Direction")

# GO terms underrepresented in forager-upregulated genes
forager_downreg_go <- hyperGTest(GSEAGOHyperGParams(name = "worker upregulated",
geneSetCollection=gsc,geneIds = intersect(forager_names,universe),
universeGeneIds=universe,ontology = "BP",pvalueCutoff = 0.05,conditional = FALSE,
testDirection = "under"))
go_forager_down<-cbind(summary(forager_upreg_go),"forager_upregulated_genes",
    "underrepresented")
colnames(go_forager_down)<-c("GOBPID","Pvalue","OddsRatio","ExpCount","Count",
    "Size","GOTerm","GeneCategory","Direction")

# GO terms overrepresneted in nurse-upregulated genes
nurse_upreg_go <- hyperGTest(GSEAGOHyperGParams(name = "worker upregulated",
    geneSetCollection=gsc,geneIds = intersect(nurse_names,universe),
    universeGeneIds=universe,ontology = "BP",pvalueCutoff = 0.05,conditional = FALSE,
  testDirection = "over"))
```

```
go_nurse_up<-cbind(summary(nurse_upreg_go),"nurse_upregulated_genes","overrepresented")
colnames(go_nurse_up)<-c("GOBPID","Pvalue","OddsRatio","ExpCount","Count","Size",
  "GOTerm","GeneCategory","Direction")

# GO terms underrepresneted in nurse-upregulated genes
nurse_downreg_go <- hyperGTest(GSEAGOHyperGParams(name = "worker upregulated",
  geneSetCollection=gsc,geneIds = intersect(nurse_names,universe),
    universeGeneIds=universe,ontology = "BP",pvalueCutoff = 0.05,conditional = FALSE,
  testDirection = "under"))
go_nurse_down<-cbind(summary(nurse_upreg_go),"nurse_upregulated_genes","underrepresented")
colnames(go_nurse_down)<-c("GOBPID","Pvalue","OddsRatio","ExpCount","Count","Size",
  "GOTerm","GeneCategory","Direction")

goanalysis<-rbind(go_forager_up,go_forager_down,go_nurse_up,go_nurse_down)
write.csv(goanalysis,file="GOanalysis.csv")

write.table(go_forager_up,file="GOanalysis.csv",append=TRUE,sep=",")
colnames(go_forager_up)<-c("GOBPID","Pvalue","OddsRatio","ExpCount","Count","Size",
  "GOTerm","GeneCategory","Direction")
```

WGCNA

```
datExpr <- data.frame(t(log(fpkm[keep,]+1,2)))
names(datExpr) <- rownames(counts[keep,])
rownames(datExpr) <- colnames(counts[keep,])
```

```
#initial exploration, barplot of mean expression per sample and sample quick dendrogram
meanExpressionByArray=apply(datExpr,1,mean,na.rm=T)
NumberMissingByArray=apply(is.na(data.frame(datExpr)),1,sum)
barplot(meanExpressionByArray,xlab="Sample",ylab="Mean expression",
        main="mean expression across samples",cex.names=0.7)
```

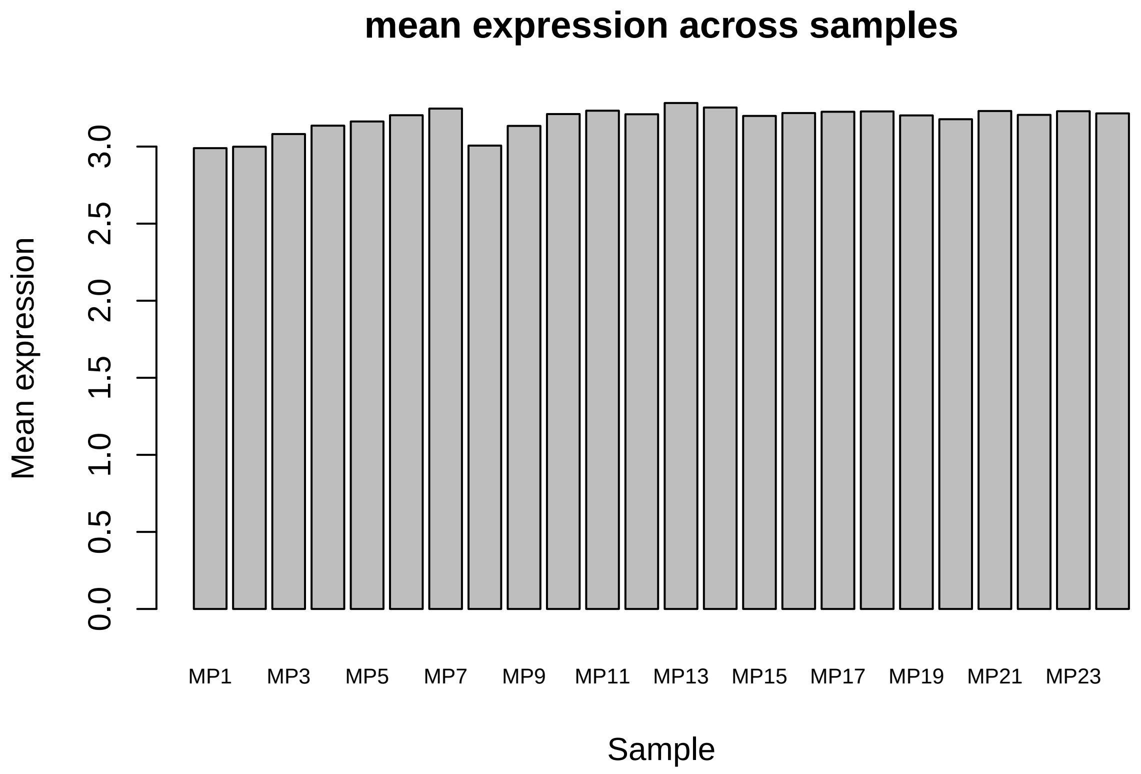


```r
plotClusterTreeSamples(datExpr=datExpr)
```

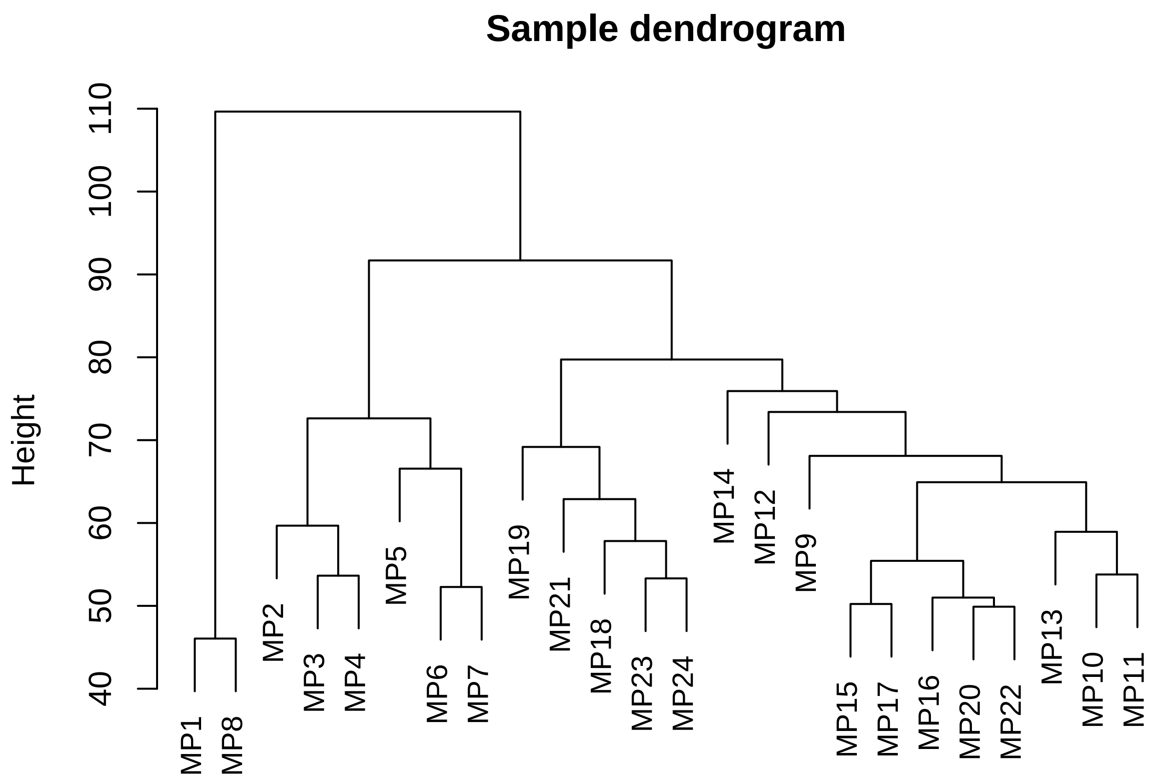


```
## NULL
```

```r
#following FemaleLiver-01-dataInput.pdf
gsg=goodSamplesGenes(datExpr,verbose=3);
```

```
##  Flagging genes and samples with too many missing values...
##   ..step 1
```

```r
gsg$allOK   # allOK
```

```
## [1] TRUE
```

```r
datExpr0=datExpr[gsg$goodSamples,gsg$goodGenes]
sampleTree=flashClust(dist(datExpr0),method="average");

#following FemaleLiver-02-networkConstr-auto.pdf
powers=c(c(1:10),seq(from=12,to=16,by=2))
allowWGCNAThreads()
```

```
## Allowing multi-threading with up to 8 threads.
```

```r
sft=pickSoftThreshold(datExpr0,powerVector=powers,verbose=5)
```

```
## pickSoftThreshold: will use block size 17371.
##  pickSoftThreshold: calculating connectivity for given powers...
##    ..working on genes 1 through 17371 of 17371
##    Power SFT.R.sq  slope truncated.R.sq mean.k. median.k. max.k.
## 1      1   0.4200  1.280          0.937  5460.0   5520.00   8290
## 2      2   0.0421 -0.193          0.776  2510.0   2460.00   5140
## 3      3   0.4260 -0.684          0.808  1380.0   1270.00   3550
## 4      4   0.6100 -0.913          0.856   843.0    712.00   2610
## 5      5   0.6900 -1.060          0.878   553.0    426.00   2000
## 6      6   0.7310 -1.140          0.892   382.0    265.00   1580
## 7      7   0.7540 -1.210          0.896   274.0    171.00   1270
## 8      8   0.7800 -1.240          0.910   203.0    113.00   1050
## 9      9   0.8030 -1.270          0.924   154.0     76.40    871
## 10    10   0.8230 -1.280          0.938   119.0     52.80    737
## 11    12   0.8490 -1.310          0.955    75.1     26.40    546
## 12    14   0.8670 -1.330          0.964    49.9     14.00    416
## 13    16   0.8670 -1.370          0.966    34.5      7.74    330
```

```r
#to graph results

par(mfrow=c(1,2));
cex=0.9;
plot(sft$fitIndices[,1],-sign(sft$fitIndices[,3])*sft$fitIndices[,2],
  xlab="Soft threshold(power)",ylab="Scale free topology model fit, signed R^2",type="n",
    main=paste("Scale independence"));
text(sft$fitIndices[,1],-sign(sft$fitIndices[,3])*sft$fitIndices[,2],
    labels=powers,cex=1,col="red");
abline(h=0.80,col="red")
plot(sft$fitIndices[,1],sft$fitIndices[,5],
    xlab="Soft threshold(power)",ylab="Mean connectivity",type="n",
    main=paste("Mean connectivity"))
text(sft$fitIndices[,1],sft$fitIndices[,5],labels=powers,cex=1,col="red")
```

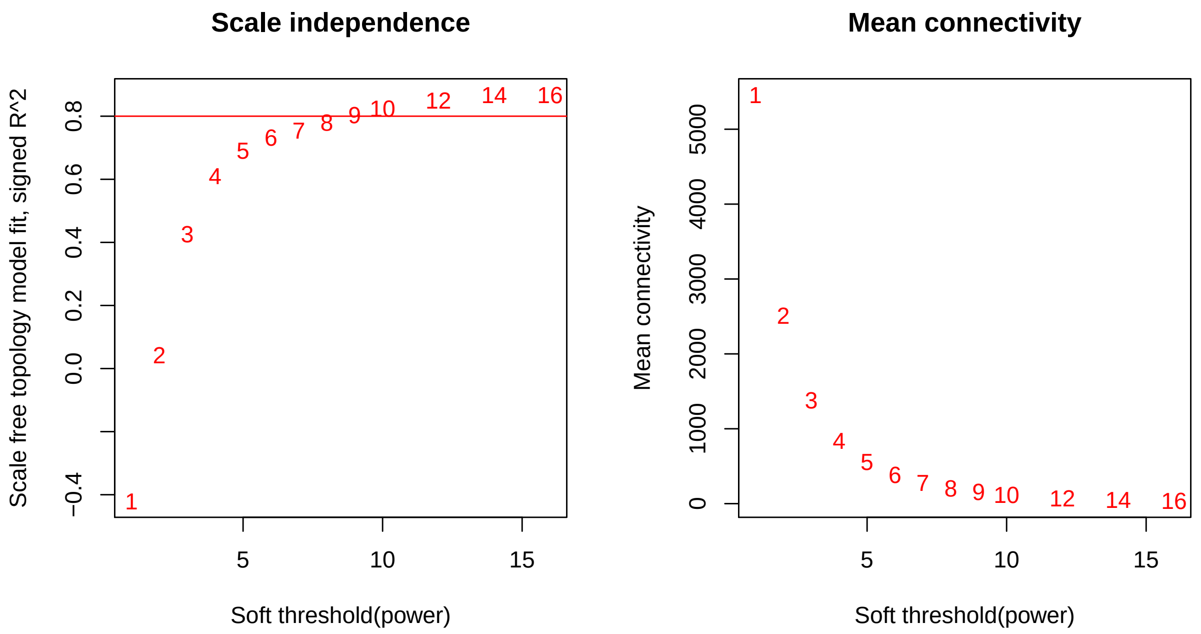

Power 9 is greater than .8, so we'll use it.

```
adjacency = adjacency(datExpr, power = 9,type="signed")
TOM = TOMsimilarity(adjacency,TOMType="signed")
```

```
datt <- datExpr

# Call the hierarchical clustering function
geneTree = flashClust(as.dist(1-TOM), method = "average");

# set the minimum module size to something relatively large
minModuleSize = 30;

# Module identification using dynamic tree cut:
dynamicMods = cutreeDynamic(dendro = geneTree, distM = 1-TOM, deepSplit = 2,
  pamRespectsDendro = FALSE, minClusterSize = minModuleSize);
```

```
##  ..cutHeight not given, setting it to 0.983  ===>  99% of the (truncated) height range in dendro.
##  ..done.
```

```
table(dynamicMods)
```

```
## dynamicMods
##    1    2    3    4    5    6    7    8    9   10   11   12   13   14   15 
## 3039 1966 1873 1653 1206 1179  598  554  476  459  407  332  285  284  278 
##   16   17   18   19   20   21   22   23   24   25   26   27   28   29   30 
##  246  223  217  194  193  158  127  126  125  120  115  113  106   98   90 
##   31   32   33   34   35   36   37 
##   88   83   81   81   76   74   48
```

```r
dynamicColors = labels2colors(dynamicMods)

# Calculate eigengenes
MEList = moduleEigengenes(datt, colors = dynamicColors)
MEs = MEList$eigengenes

# Calculate dissimilarity of module eigengenes
METree = flashClust(as.dist(1-cor(MEs)), method = "average");
plot(METree, main = "Clustering of module eigengenes",xlab = "", sub = "")
MEDissThres = 0.25

# Plot the cut line into the dendrogram
abline(h=MEDissThres, col = "red")
```

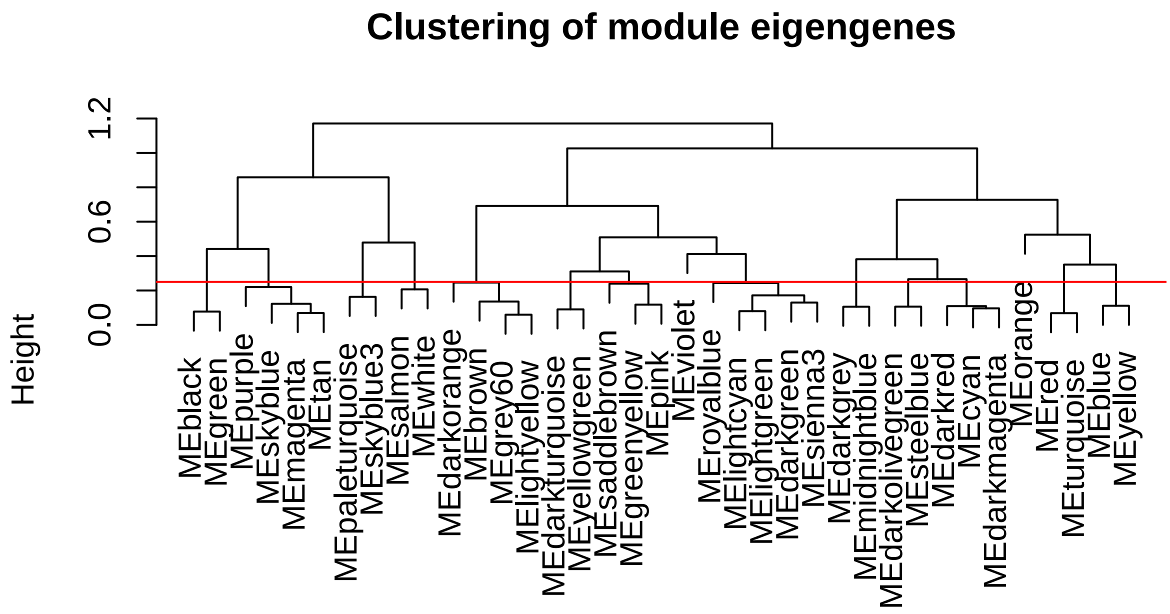


```r
# Call an automatic merging function
merge <- mergeCloseModules(datt, dynamicColors, cutHeight = MEDissThres, verbose = 0)

# The merged module colors
mergedColors = merge$colors

# Eigengenes of the new merged modules:
mergedMEs = merge$newMEs

# plotting the cluster dendrogram
plotDendroAndColors(geneTree, cbind(dynamicColors, mergedColors),
```

```
c("Dynamic Tree Cut", "Merged dynamic"),
dendroLabels = FALSE, hang = 0.03,
addGuide = TRUE, guideHang = 0.05)
```

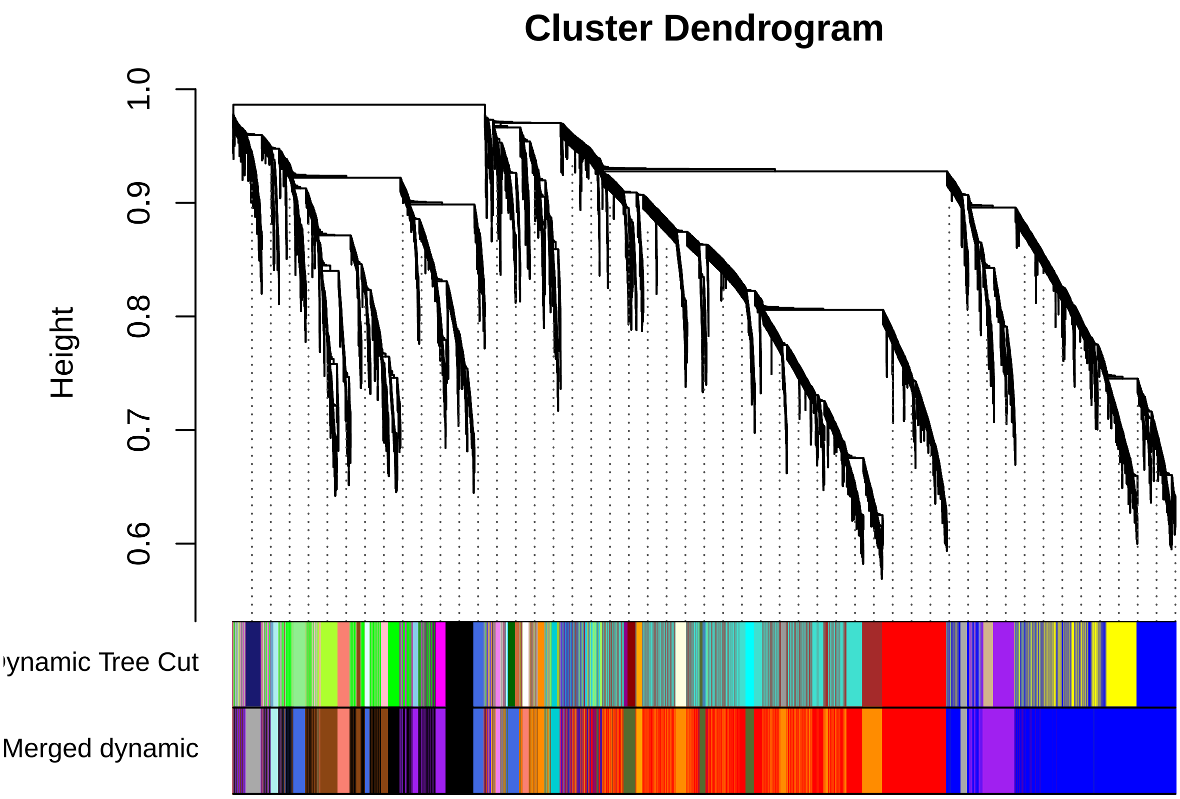


```
# Rename to moduleColors
moduleColors = mergedColors

# Construct numerical labels corresponding to the colors
colorOrder = c("grey", standardColors(50));
moduleLabels = match(moduleColors, colorOrder)-1;
MEs = mergedMEs;

# Define numbers of genes and samples
nGenes = ncol(datt);
nSamples = nrow(datt);

# Recalculate MEs with color labels
invisible(MEs0 <- moduleEigengenes(datt, moduleColors)$eigengenes)
MEs = orderMEs(MEs0)
MEs<-MEs[,order(names(MEs))]

# correlations of genes with eigengenes
moduleGeneCor=cor(MEs,datt)
moduleGenePvalue = corPvalueStudent(moduleGeneCor, nSamples);
```

```r
# how many genes in each module?
table(moduleColors)
```

```
## moduleColors
##          black           blue       darkgrey darkolivegreen     darkorange
##           1804           3619            403            694           2405
##  darkturquoise         orange  paleturquoise         purple            red
##            200            120            136           1373           4218
##      royalblue    saddlebrown         salmon         violet
##            859           1059            398             83
```

```r
connectivity <- intramodularConnectivity(adjacency,merge$colors)
```

go-term enrichment in modules, output in table

```r
modules<-c("saddlebrown","salmon","black","paleturquoise","royalblue","violet",
"darkturquoise","darkorange","darkolivegreen","red","orange","purple","darkgrey","blue")

for (i in 1:length(modules)) {
  module_genes <- rownames(counts[keep,])[moduleColors == modules[i]]
  module_upreg <- hyperGTest(GSEAGOHyperGParams(name = paste(modules[i],"upregulated"),
  geneSetCollection=gsc,geneIds = intersect(module_genes,universe),
  universeGeneIds=universe,ontology = "BP",pvalueCutoff = 0.05,conditional = FALSE,
  testDirection = "over"))
module_upreg1<-cbind(summary(module_upreg),i,modules[i])
colnames(module_upreg1)<-c("GOBPID","Pvalue","OddsRatio","ExpCount","Count",
  "Size","GOTerm","ModuleNumber","ModuleColor")
  write.table(module_upreg1,file="GOmodule.csv",append=TRUE,sep=",",row.names=FALSE)
}
```

module-trait correlations

```r
plot_order <-  c(8,1,5,6,7,2,3,4,9)
traits = cbind(subset(design, select = -c(troph,groom, carb,prot)),forager=design[,"prot"]+
  design[,"carb"])[,plot_order]

moduleTraitCor = cor(MEs, traits, use = "p")
moduleTraitPvalue = p.adjust(corPvalueStudent(moduleTraitCor, nSamples=nrow(MEs)),method="fdr")

textMatrix = paste(signif(moduleTraitCor, 2), "\n(",
signif(moduleTraitPvalue, 1), ")", sep = "");
dim(textMatrix) = dim(moduleTraitCor)
par(mar = c(6, 8.5, 3, 3));

# Display the correlation values within a heatmap plot
labeledHeatmap(Matrix = moduleTraitCor,
xLabels = colnames(moduleTraitCor),
yLabels = names(MEs),
```

```
ySymbols = names(MEs),
colorLabels = FALSE,
colors = blueWhiteRed(50),
textMatrix = textMatrix,
setStdMargins = FALSE,
cex.text = .5,
zlim = c(-1,1),
main = paste("Module-trait relationships"))
```

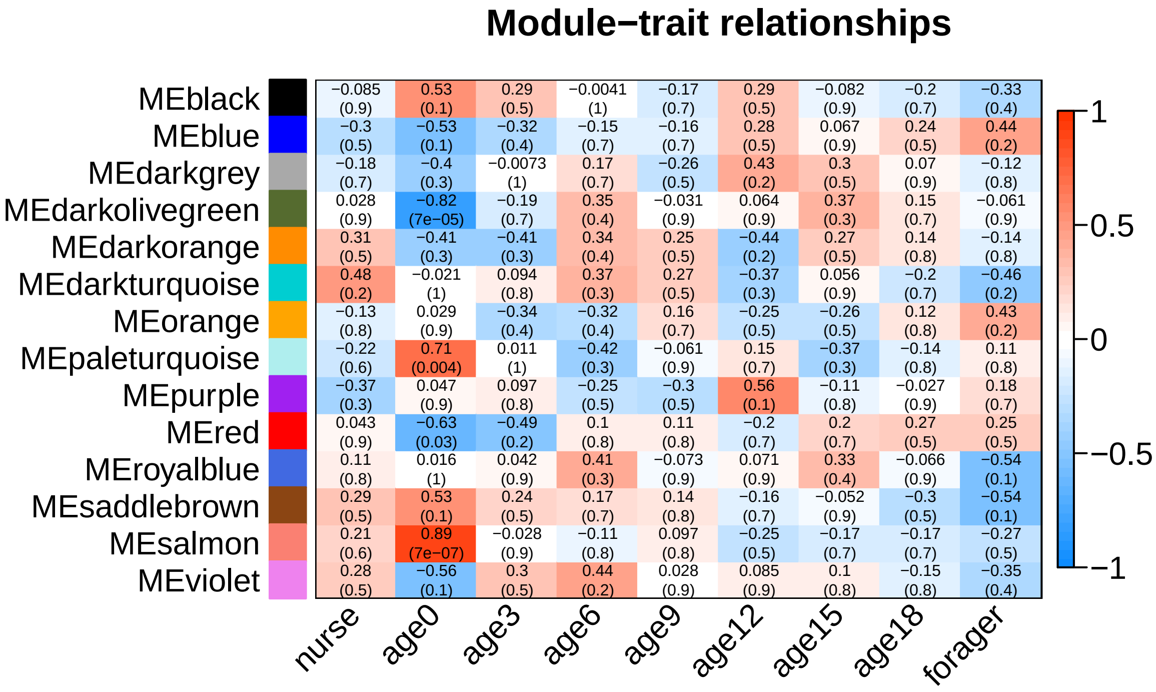


```
mycols <- colorpanel(n=19,low="white",high="darkgreen")
heatmap.2(moduleTraitCor,key=FALSE,trace="none",col=mycols,Colv=NULL,labCol=colnames(traits),
density.info="none", hclustfun=function(c){hclust(c, method="complete")},margins = c(10, 4),
RowSideColors=gsub("ME","",names(MEs)),labRow=table(moduleColors))
```

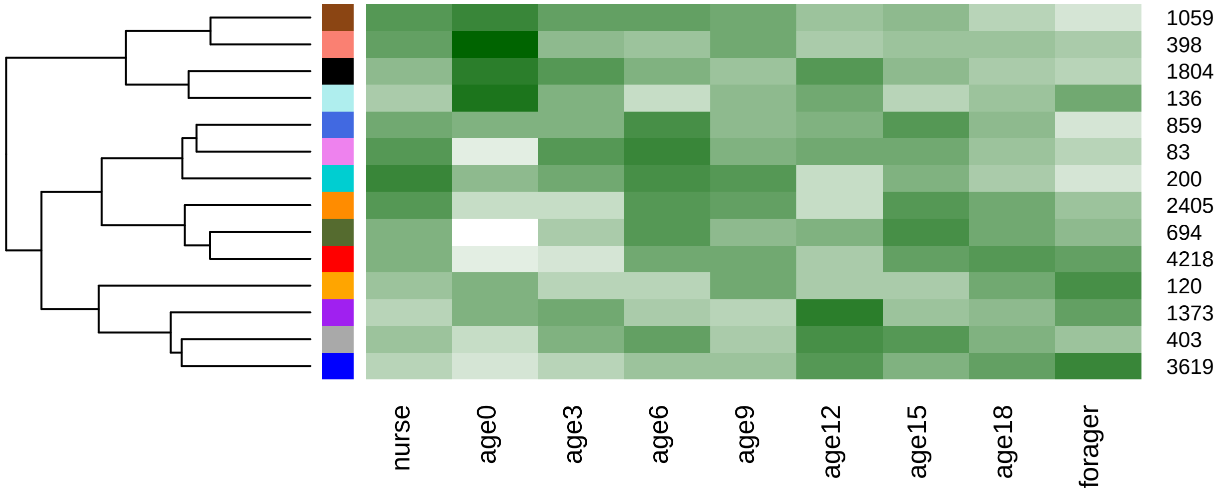

Plotting module-age correlation coefficient over time

```
moduleTraitCor_stack <- melt(moduleTraitCor)
colnames(moduleTraitCor_stack) <- c("module","stage","corr")
moduleTraitCor_stack$stage <- factor(moduleTraitCor_stack$stage,unique(moduleTraitCor_stack[,2]))
ggplot(subset(moduleTraitCor_stack, stage !="nurse" & stage != "forager"),aes(x=stage,y=corr,
  color=module,group=module)) + geom_line()+theme_bw()
```

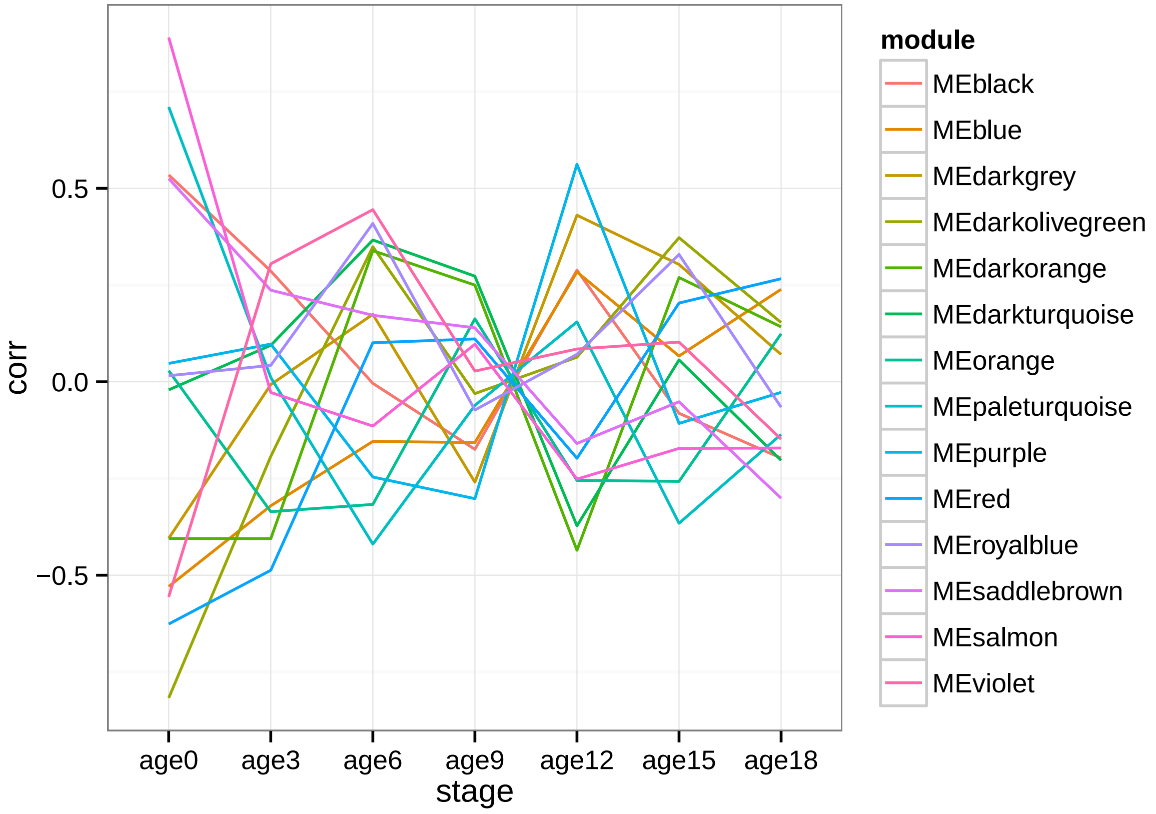


```
pvals <-  c()
for (i in 1:nrow(moduleTraitCor))
    pvals <- c(pvals , cor.test(t(moduleTraitCor)[-c(1,9),i],seq(0,18,3))$p.value)
unique(names(MEs))[p.adjust(pvals,method="fdr")<.05]
```

```
## [1] "MEblue"        "MEsaddlebrown"
```

```
#the saddelbrown and blue modules are significantly correlated with age
```

Connectivity and evolution

This section examines whether connectivity plays a role in the evolution of genes.

connectivity vs selection

Set up a data frame with connectivities and rates, and see whther total connectivity affects rates of evolution

```
genes_with_rates <- intersect(rownames(connectivity),manfredini$mp_id)
connectivity_rate <- cbind(manfredini[genes_with_rates,"rate"],
  connectivity[rownames(connectivity) %in% genes_with_rates,])
colnames(connectivity_rate) <- c("rate",names(connectivity))
#add task information
```

```r
connectivity_rate$task <- "all others"
connectivity_rate[forager_names,"task"] <- "forager"
connectivity_rate[nurse_names,"task"] <- "nurse"
connectivity_rate$task <- factor(connectivity_rate$task, levels=c("all others","nurse","forager"))

#evolutionary rate and connectivity
with(connectivity_rate,cor.test(rate,kTotal,method="spearman"))
```

```
##
##  Spearman's rank correlation rho
##
## data:  rate and kTotal
## S = 8.002e+10, p-value = 3.125e-14
## alternative hypothesis: true rho is not equal to 0
## sample estimates:
##      rho
## -0.08686
```

There is a negative correlation between total connectivity and evolutionary rate, which is typical

expression and connectivity

average fpkm vs connectivity

```r
cor.test(rowMeans(fpkm)[rownames(connectivity_rate)],connectivity_rate$kTotal,method="spearman")
```

```
##
##  Spearman's rank correlation rho
##
## data:  rowMeans(fpkm)[rownames(connectivity_rate)] and connectivity_rate$kTotal
## S = 5.119e+10, p-value < 2.2e-16
## alternative hypothesis: true rho is not equal to 0
## sample estimates:
##    rho
## 0.3047
```

Highly connected genes have higher rates of expression, which is also expected.

```r
kruskalmc(kTotal ~ task, data = connectivity_rate)
```

```
## Multiple comparison test after Kruskal-Wallis
## p.value: 0.05
## Comparisons
##                    obs.dif critical.dif difference
## all others-nurse      1011        235.4       TRUE
## all others-forager    1971        215.6       TRUE
## nurse-forager         2982        305.4       TRUE
```

All tasks differ in connectivity.

Model for joint effects of expression and network on evolution

```
connectivity_rate$fpkm <- fpkm_task[rownames(connectivity_rate),"fpkm"]
connectivity_rate$task <- fpkm_task[rownames(connectivity_rate),"task"]
connectivity_rate$task <- factor(connectivity_rate$task, levels=c("nde","nurse","forager"))

connectivity_rate$logFC <- et_nurse_forager$table[rownames(connectivity_rate),"logFC"]

form1 <- log(rate) ~ factor(task)
form2 <- log(rate) ~ log(fpkm)*factor(task)+log(kTotal)*factor(task)

summary(rate1.lm <- lm(form1, data=subset(connectivity_rate,rate<10 )))
```

```
##
## Call:
## lm(formula = form1, data = subset(connectivity_rate, rate < 10))
##
## Residuals:
##    Min     1Q Median     3Q    Max
## -4.679 -0.555  0.239  0.787  4.053
##
## Coefficients:
##                       Estimate Std. Error t value Pr(>|t|)
## (Intercept)            -2.3850     0.0148 -160.73   <2e-16 ***
## factor(task)nurse       0.1564     0.0531    2.94   0.0032 **
## factor(task)forager     0.0815     0.0487    1.67   0.0944 .
## ---
## Signif. codes:  0 '***' 0.001 '**' 0.01 '*' 0.05 '.' 0.1 ' ' 1
##
## Residual standard error: 1.19 on 7596 degrees of freedom
## Multiple R-squared:  0.00141,    Adjusted R-squared:  0.00114
## F-statistic: 5.35 on 2 and 7596 DF,  p-value: 0.00475
```

```
summary(rate2.lm <-lm(form2, data=subset(connectivity_rate,rate<10 )))
```

```
##
## Call:
## lm(formula = form2, data = subset(connectivity_rate, rate < 10))
##
## Residuals:
##    Min     1Q Median     3Q    Max
## -4.893 -0.566  0.238  0.776  4.108
##
## Coefficients:
##                                Estimate Std. Error t value Pr(>|t|)
## (Intercept)                   -1.684419   0.121585  -13.85  < 2e-16 ***
## log(fpkm)                     -0.099207   0.013370   -7.42  1.3e-13 ***
## factor(task)nurse              0.508210   0.772553    0.66  0.51067
## factor(task)forager           -0.049509   0.841200   -0.06  0.95307
## log(kTotal)                   -0.074624   0.020250   -3.69  0.00023 ***
## log(fpkm):factor(task)nurse    0.071554   0.037099    1.93  0.05381 .
## log(fpkm):factor(task)forager  0.081432   0.045733    1.78  0.07501 .
```

```
## factor(task)nurse:log(kTotal)   -0.086372   0.128836   -0.67  0.50262
## factor(task)forager:log(kTotal) -0.000486   0.124833    0.00  0.99690
## ---
## Signif. codes:  0 '***' 0.001 '**' 0.01 '*' 0.05 '.' 0.1 ' ' 1
##
## Residual standard error: 1.18 on 7590 degrees of freedom
## Multiple R-squared:  0.0144, Adjusted R-squared:  0.0134
## F-statistic: 13.9 on 8 and 7590 DF,  p-value: <2e-16
```

```
# verifying results using bootstrap
bs <- function(formula, data, indices) {
  d <- data[indices,] # allows boot to select sample
  glm(formula, data=d)
  fit <- lm(formula, data=d)
  return(coef(fit))
}
results <- boot(data=connectivity_rate, statistic=bs, R=1000, formula=form1)

for (i in 2:length(rate1.lm$coefficients)) {
  bci <- boot.ci(results, type="basic", index=i)
  print(sprintf("%s,%.4f,%.4f,%.4f", names(rate1.lm$coefficients)[i],
              results$t0[i], bci$basic[4], bci$basic[5]))
}
```

```
## [1] "factor(task)nurse,0.1418,0.0414,0.2523"
## [1] "factor(task)forager,0.0857,-0.0103,0.1796"
```

```
results <- boot(data=connectivity_rate, statistic=bs, R=1000, formula=form2)

for (i in 2:length(rate2.lm$coefficients)) {
  bci <- boot.ci(results, type="basic", index=i)
  print(sprintf("%s,%.4f,%.4f,%.4f", names(rate2.lm$coefficients)[i],results$t0[i], bci$basic[4], bci$ba
}
```

```
## [1] "log(fpkm),-0.1037,-0.1330,-0.0726"
## [1] "factor(task)nurse,0.4304,-1.1489,2.1010"
## [1] "factor(task)forager,-0.0100,-1.5854,1.6418"
## [1] "log(kTotal),-0.0830,-0.1227,-0.0432"
## [1] "log(fpkm):factor(task)nurse,0.0761,0.0017,0.1473"
## [1] "log(fpkm):factor(task)forager,0.1085,0.0057,0.2258"
## [1] "factor(task)nurse:log(kTotal),-0.0780,-0.3542,0.1846"
## [1] "factor(task)forager:log(kTotal),-0.0152,-0.2581,0.2233"
```

connectivity and context of expression

Here, we are interested in the regulatory context of forager- and nurse-expressed genes. This incudes *all* genes, not just those with evolutionary rate estimates.

```
connectivity$task <- "all others"
connectivity[forager_names,"task"] <- "forager"
connectivity[nurse_names,"task"] <- "nurse"
```

```r
connectivity$task <- factor(connectivity$task, levels=c("all others","nurse","forager"))

kruskalmc(kTotal ~ task, data = connectivity)
```

```
## Multiple comparison test after Kruskal-Wallis
## p.value: 0.05
## Comparisons
##                      obs.dif critical.dif difference
## all others-nurse       830.9        353.9       TRUE
## all others-forager    5071.1        357.9       TRUE
## nurse-forager         5902.0        483.7       TRUE
```

```r
ggplot(connectivity,aes(x=factor(task),y=kTotal))+geom_boxplot(notch=TRUE)+theme_bw()+
  ylab("connectivity")+xlab("")+scale_y_log10()
```

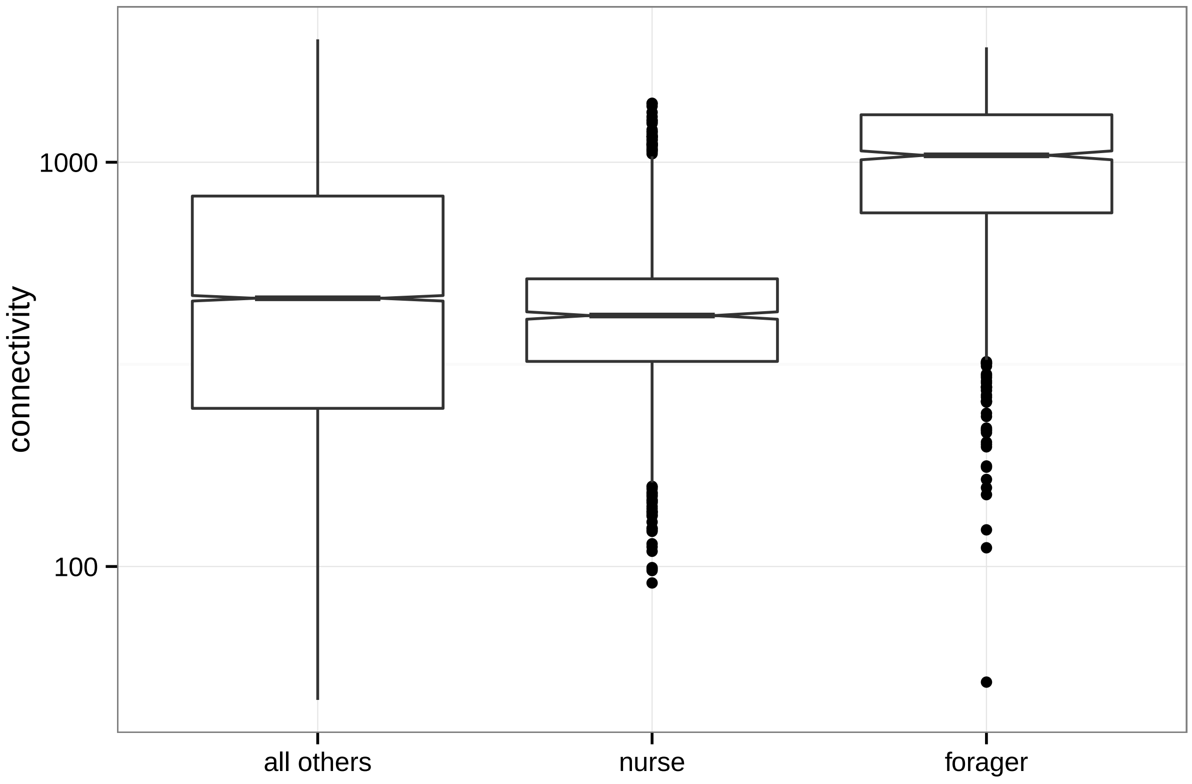

The picture is similar to what you find with genes that have rates.

Effect of connectivity on presence of S. invicta or A. mellifera ortholog

```r
connectivity$homolog <- 0
connectivity[rownames(manfredini)[rownames(manfredini) %in% rownames(connectivity)],"homolog"]<-1

kruskal.test(kTotal ~ factor(homolog), data=connectivity)
```

```
##
##  Kruskal-Wallis rank sum test
##
## data:  kTotal by factor(homolog)
## Kruskal-Wallis chi-squared = 724, df = 1, p-value < 2.2e-16
```

```
ggplot(connectivity,aes(factor(homolog),kTotal))+geom_violin()+facet_grid(.~task)+theme_bw()+
  stat_summary(fun.data="mean_cl_boot", geom="errorbar",width=0.1,colour = "red")
```

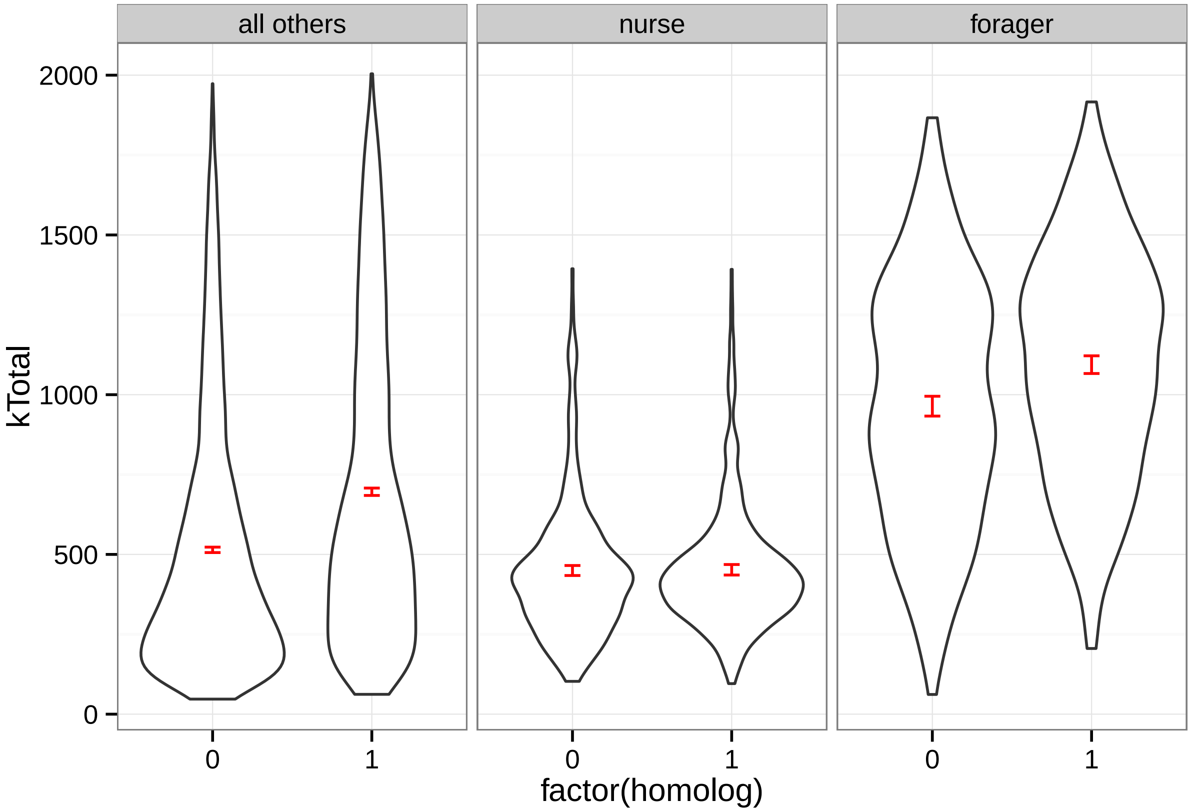


```
kruskalmc(homolog ~ task, data=connectivity)
```

```
## Multiple comparison test after Kruskal-Wallis
## p.value: 0.05
## Comparisons
##                     obs.dif critical.dif difference
## all others-nurse      36.26        353.9      FALSE
## all others-forager   950.05        357.9       TRUE
## nurse-forager        913.79        483.7       TRUE
```

```
summary(glm(log(kTotal)~factor(task)*factor(homolog),data=connectivity))
```

```
##
## Call:
## glm(formula = log(kTotal) ~ factor(task) * factor(homolog), data = connectivity)
```

```
##
## Deviance Residuals:
##     Min       1Q   Median       3Q      Max
## -2.8214  -0.4966   0.0333   0.5390   1.6387
##
## Coefficients:
##                                     Estimate Std. Error t value Pr(>|t|)
## (Intercept)                          5.94894    0.00805  738.90  < 2e-16
## factor(task)nurse                    0.05459    0.02908    1.88  0.06046
## factor(task)forager                  0.81926    0.03237   25.31  < 2e-16
## factor(homolog)1                     0.33506    0.01227   27.30  < 2e-16
## factor(task)nurse:factor(homolog)1   -0.30489    0.04412   -6.91    5e-12
## factor(task)forager:factor(homolog)1 -0.17047    0.04439   -3.84  0.00012
##
## (Intercept)                          ***
## factor(task)nurse                    .
## factor(task)forager                  ***
## factor(homolog)1                     ***
## factor(task)nurse:factor(homolog)1   ***
## factor(task)forager:factor(homolog)1 ***
## ---
## Signif. codes:  0 '***' 0.001 '**' 0.01 '*' 0.05 '.' 0.1 ' ' 1
##
## (Dispersion parameter for gaussian family taken to be 0.5503)
##
##     Null deviance: 10652.4  on 17370  degrees of freedom
## Residual deviance:  9556.2  on 17365  degrees of freedom
## AIC: 38930
##
## Number of Fisher Scoring iterations: 2
```

connectivity and expression is higher for forager-upregulated genes and for genes with S. invicta orthologs. The connectivity of forager- and nurse-upregulated genes is less affected by whether the gene has a S. invicta ortholog than non-differentially expressed genes

```
connectivity$beehomolog <- 0
connectivity[hbee$mp[hbee$mp %in% rownames(connectivity)],"beehomolog"] <- 1

kruskal.test(kTotal ~ factor(beehomolog), data=connectivity)
```

```
##
##  Kruskal-Wallis rank sum test
##
## data:  kTotal by factor(beehomolog)
## Kruskal-Wallis chi-squared = 1215, df = 1, p-value < 2.2e-16
```

```
ggplot(connectivity,aes(factor(beehomolog),kTotal))+geom_violin()+facet_grid(.~task)+theme_bw()+
  stat_summary(fun.data="mean_cl_boot", geom="errorbar",width=0.1,colour = "red")
```

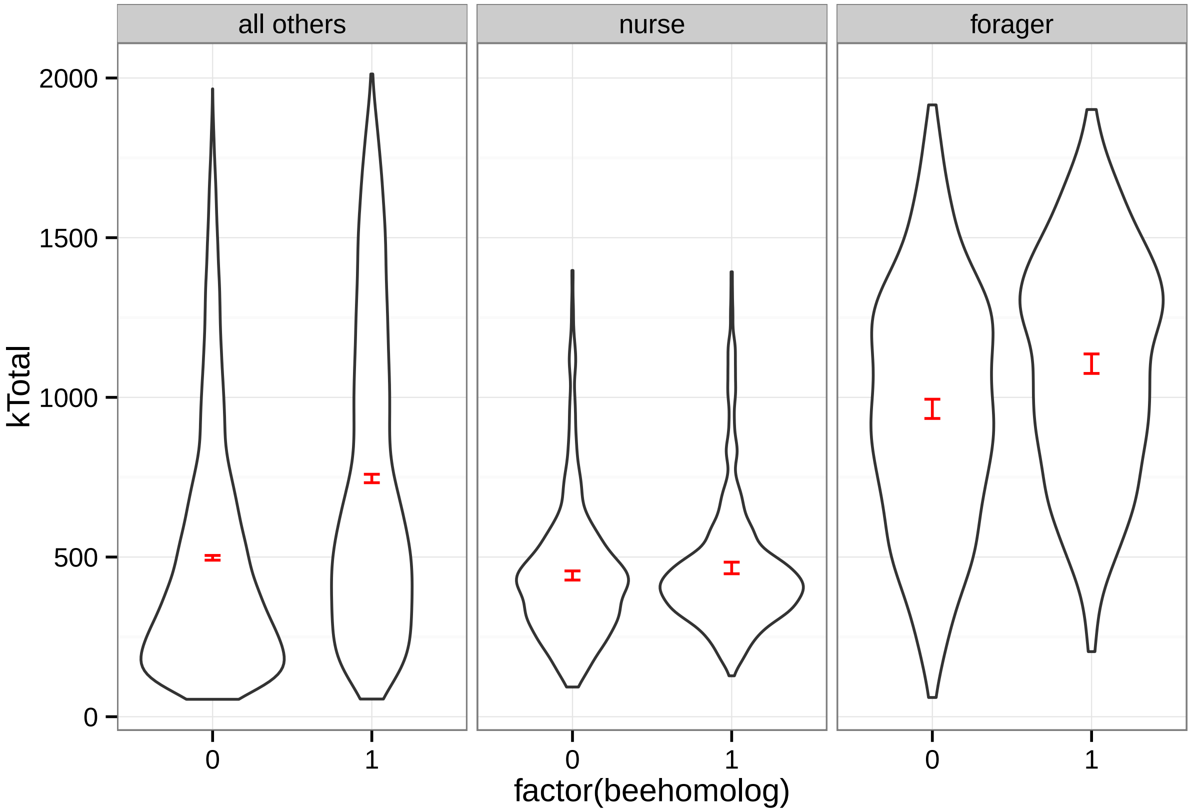


```
kruskalmc(beehomolog ~ task, data=connectivity)
```

```
## Multiple comparison test after Kruskal-Wallis
## p.value: 0.05
## Comparisons
##                      obs.dif critical.dif difference
## all others-nurse       8.828        353.9      FALSE
## all others-forager 982.999        357.9       TRUE
## nurse-forager      974.172        483.7       TRUE
```

```
summary(glm(log(kTotal)~factor(task)*factor(beehomolog),data=connectivity))
```

```
##
## Call:
## glm(formula = log(kTotal) ~ factor(task) * factor(beehomolog),
##     data = connectivity)
##
## Deviance Residuals:
##     Min       1Q   Median       3Q      Max
## -2.8234  -0.4914   0.0307   0.5359   1.6672
##
## Coefficients:
##                                         Estimate Std. Error t value
## (Intercept)                               5.9205     0.0076  778.59
```

```
## factor(task)nurse                            0.0669     0.0274    2.44
## factor(task)forager                          0.8497     0.0304   27.94
## factor(beehomolog)1                          0.4517     0.0123   36.73
## factor(task)nurse:factor(beehomolog)1       -0.3753     0.0442   -8.48
## factor(task)forager:factor(beehomolog)1     -0.2764     0.0436   -6.34
##                                            Pr(>|t|)
## (Intercept)                                 < 2e-16 ***
## factor(task)nurse                             0.015 *
## factor(task)forager                         < 2e-16 ***
## factor(beehomolog)1                         < 2e-16 ***
## factor(task)nurse:factor(beehomolog)1       < 2e-16 ***
## factor(task)forager:factor(beehomolog)1     2.4e-10 ***
## ---
## Signif. codes:  0 '***' 0.001 '**' 0.01 '*' 0.05 '.' 0.1 ' ' 1
##
## (Dispersion parameter for gaussian family taken to be 0.5324)
##
##     Null deviance: 10652.4  on 17370  degrees of freedom
## Residual deviance:  9245.6  on 17365  degrees of freedom
## AIC: 38356
##
## Number of Fisher Scoring iterations: 2
```

similarly, connectivity is higher for forager-upregulated genes and for genes with A. mellifera orthologs. The connectivity of forager- and nurse-upregulated genes is less affected by whether the gene has a A. mellifera ortholog than non-differentially expressed genes

```
connectivity$fpkm <- rowMeans(fpkm[keep,])
summary(glm(homolog~fpkm*factor(task)+kTotal*factor(task),family=quasibinomial,data=connectivity))
```

```
##
## Call:
## glm(formula = homolog ~ fpkm * factor(task) + kTotal * factor(task),
##     family = quasibinomial, data = connectivity)
##
## Deviance Residuals:
##    Min      1Q  Median      3Q     Max
## -1.637  -1.030  -0.892   1.270   2.802
##
## Coefficients:
##                           Estimate Std. Error t value Pr(>|t|)
## (Intercept)              -8.53e-01   2.89e-02  -29.50  < 2e-16 ***
## fpkm                     -1.25e-05   3.06e-05   -0.41   0.6831
## factor(task)nurse         5.59e-01   1.40e-01    4.00  6.4e-05 ***
## factor(task)forager      -1.00e-03   1.76e-01   -0.01   0.9955
## kTotal                    9.58e-04   3.92e-05   24.46  < 2e-16 ***
## fpkm:factor(task)nurse   -1.37e-05   5.33e-05   -0.26   0.7974
## fpkm:factor(task)forager -6.69e-04   2.87e-04   -2.33   0.0198 *
## factor(task)nurse:kTotal -8.82e-04   2.78e-04   -3.17   0.0015 **
## factor(task)forager:kTotal  6.29e-05   1.66e-04    0.38   0.7050
## ---
## Signif. codes:  0 '***' 0.001 '**' 0.01 '*' 0.05 '.' 0.1 ' ' 1
##
```

```
## (Dispersion parameter for quasibinomial family taken to be 1.002)
##
##     Null deviance: 23817  on 17370  degrees of freedom
## Residual deviance: 23088  on 17362  degrees of freedom
## AIC: NA
##
## Number of Fisher Scoring iterations: 4
```

The likelihood of a gene to have a S. invicta ortholog is most strongly determined by its connectivity.

```
summary(glm(beehomolog~fpkm*factor(task)+kTotal*factor(task),family=quasibinomial,
            data=connectivity))
```

```
##
## Call:
## glm(formula = beehomolog ~ fpkm * factor(task) + kTotal * factor(task),
##     family = quasibinomial, data = connectivity)
##
## Deviance Residuals:
##    Min      1Q  Median      3Q     Max
## -1.755  -0.943  -0.779   1.238   1.902
##
## Coefficients:
##                              Estimate Std. Error t value Pr(>|t|)
## (Intercept)                 -1.28e+00   3.05e-02  -41.82  < 2e-16 ***
## fpkm                        -4.88e-06   2.88e-05   -0.17   0.8654
## factor(task)nurse            5.63e-01   1.42e-01    3.95  7.7e-05 ***
## factor(task)forager          1.50e-01   1.79e-01    0.84   0.4024
## kTotal                       1.31e-03   4.05e-05   32.23  < 2e-16 ***
## fpkm:factor(task)nurse      -1.33e-05   4.41e-05   -0.30   0.7624
## fpkm:factor(task)forager    -8.88e-04   3.44e-04   -2.58   0.0098 **
## factor(task)nurse:kTotal    -7.75e-04   2.80e-04   -2.76   0.0057 **
## factor(task)forager:kTotal -1.90e-04   1.67e-04   -1.14   0.2553
## ---
## Signif. codes:  0 '***' 0.001 '**' 0.01 '*' 0.05 '.' 0.1 ' ' 1
##
## (Dispersion parameter for quasibinomial family taken to be 0.9974)
##
##     Null deviance: 23238  on 17370  degrees of freedom
## Residual deviance: 22001  on 17362  degrees of freedom
## AIC: NA
##
## Number of Fisher Scoring iterations: 4
```

similarly, the likelihood of a gene to have a A. mellifera ortholog is most strongly determined by its connectivity.

```
summary(beehomolog~task,data=connectivity)
```

```
## beehomolog    N=17371
##
## +--------+----------+-----+----------+
## |        |          |N    |beehomolog|
```

```
## +--------+----------+-----+----------+
## |task    |all others|14907|0.3823    |
## |        |nurse     | 1247|0.3833    |
## |        |forager   | 1217|0.4955    |
## +--------+----------+-----+----------+
## |Overall|          |17371|0.3903    |
## +--------+----------+-----+----------+
```

```
with(connectivity,table(beehomolog,task))
```

```
##           task
## beehomolog all others nurse forager
##          0       9208   769     614
##          1       5699   478     603
```

```
chisq.test(with(connectivity,table(beehomolog,task)))
```

```
##
##  Pearson's Chi-squared test
##
## data:  with(connectivity, table(beehomolog, task))
## X-squared = 60.84, df = 2, p-value = 6.153e-14
```

49% of forager-upregulated genes have honey bee orthologs, compared to 38% for nurse-upregulated and non-differentially expressed genes

```
summary(homolog~task,data=connectivity)
```

```
## homolog    N=17371
##
## +--------+----------+-----+-------+
## |        |          |N    |homolog|
## +--------+----------+-----+-------+
## |task    |all others|14907|0.4305 |
## |        |nurse     | 1247|0.4346 |
## |        |forager   | 1217|0.5399 |
## +--------+----------+-----+-------+
## |Overall|          |17371|0.4384 |
## +--------+----------+-----+-------+
```

```
with(connectivity,table(homolog,task))
```

```
##        task
## homolog all others nurse forager
##       0       8490   705     560
##       1       6417   542     657
```

```
chisq.test(with(connectivity,table(homolog,task)))
```

```
##
##  Pearson's Chi-squared test
##
## data:  with(connectivity, table(homolog, task))
## X-squared = 54.76, df = 2, p-value = 1.288e-12
```

similarly, forager-upregulated genes are much more likely to have fire ant orthologs (54%) compared to nurse-upregulated and non-differentially expressed genes (43%)

```
con<-as.data.table(connectivity)
con$hom<-"none"
con[homolog=="1" & beehomolog=="1", hom :="both"]
con[homolog=="1" & beehomolog=="0", hom :="Sinv only"]
con[homolog=="0" & beehomolog=="1", hom :="Amel only"]
con[homolog=="0" & beehomolog=="0", hom :="neither"]
con$hom<-factor(con$hom,levels=c("both","neither","Amel only","Sinv only"))
```

```
with(con,table(hom,task))
```

```
##            task
## hom          all others nurse forager
##   both             4739   395     521
##   neither          7530   622     478
##   Amel only         960    83      82
##   Sinv only        1678   147     136
```

```
chisq.test(with(con,table(hom,task)))
```

```
##
##  Pearson's Chi-squared test
##
## data:  with(con, table(hom, task))
## X-squared = 71.42, df = 6, p-value = 2.093e-13
```

forager-upregulated genes are more likely to have both fire ant and honey bee orthologs

Overall, highly connected genes are more likely to have a ortholog, and correspondingly forager-upregulated genes are more conserved.

Summary plots

```
#connectivity vs. honey bee ortholog
fmt <- function() function(x) formatC(signif(x,digits=3), digits=2,format="fg")
connectivity$task<-factor(connectivity$task,labels=c("NDE","Nurse","Forager"))
connectivity$beehomolog<-factor(connectivity$beehomolog,labels=c("No","Yes"))
beeortholog_plot<-ggplot(connectivity,aes(factor(beehomolog),kTotal,fill=task,alpha=0.2))+
  geom_violin()+facet_grid(.~task)+theme_bw()+
  ylab("Connectivity (kTotal)")+scale_y_log10()+
  xlab("Honey bee ortholog")+
```

```
  scale_fill_manual(values=c("grey","blue","red"))+
  theme(legend.position="none")+theme(plot.margin=unit(c(0,0.5,0.5,0.5), "cm"),
  axis.text.y = element_text(size=14),axis.text.x = element_text(size=14),
  axis.title.y = element_text(size=20),axis.title.x = element_text(size=20))+
  ggtitle("")+
  theme(strip.text.x = element_text(size=16),
        strip.background = element_rect(fill="cornsilk1"))+
  stat_summary(fun.data="mean_cl_boot", geom="errorbar",width=0.15,alpha=1,size=1,colour = "black")
beeortholog_plot
```

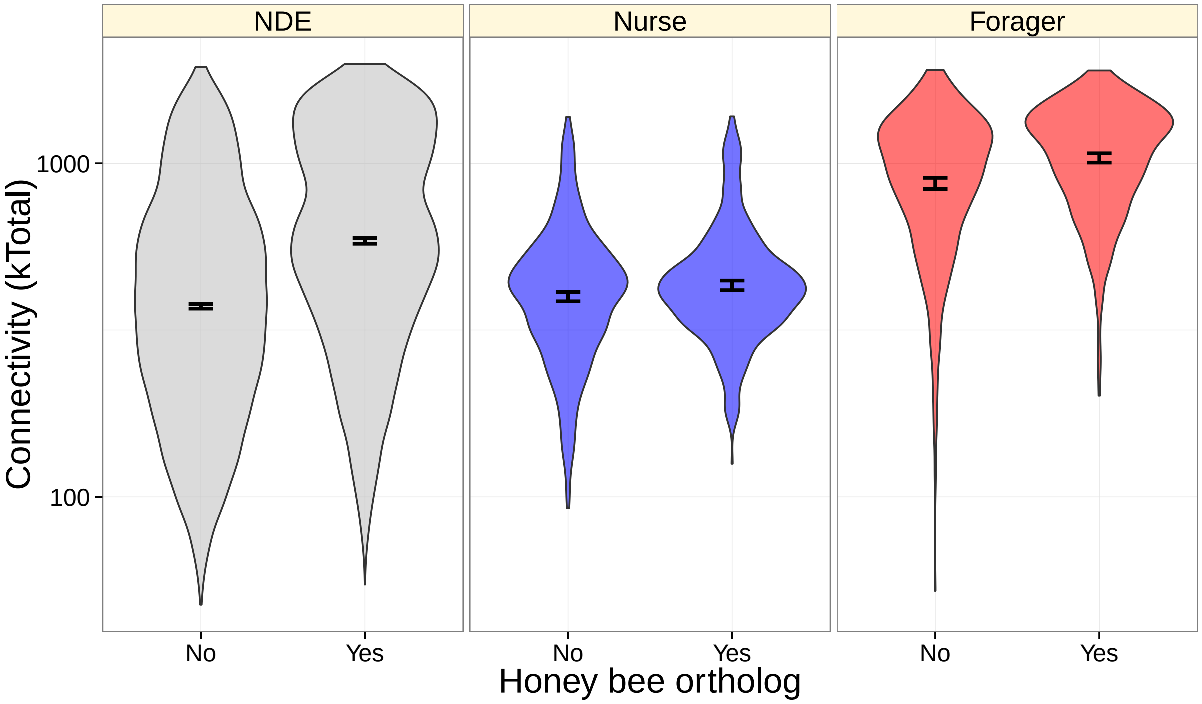


```
#connectivity vs. fire ant ortholog
connectivity$homolog<-factor(connectivity$homolog,labels=c("No","Yes"))
antortholog_plot<-ggplot(connectivity,aes(factor(homolog),kTotal,fill=task,alpha=0.2))+
  geom_violin()+facet_grid(.~task)+theme_bw()+
  ylab("Connectivity (kTotal)")+scale_y_log10()+
  xlab("Fire ant ortholog")+
  scale_fill_manual(values=c("grey","blue","red"))+
  theme(legend.position="none")+theme(plot.margin=unit(c(0,0.5,0.5,0.5), "cm"),
  axis.text.y = element_text(size=14),axis.text.x = element_text(size=14),
  axis.title.y = element_text(size=20),axis.title.x = element_text(size=20))+
  ggtitle("")+
  theme(strip.text.x = element_text(size=16),
        strip.background = element_rect(fill="cornsilk1"))+
  stat_summary(fun.data="mean_cl_boot", geom="errorbar",width=0.15,alpha=1,size=1,colour = "black")
antortholog_plot
```

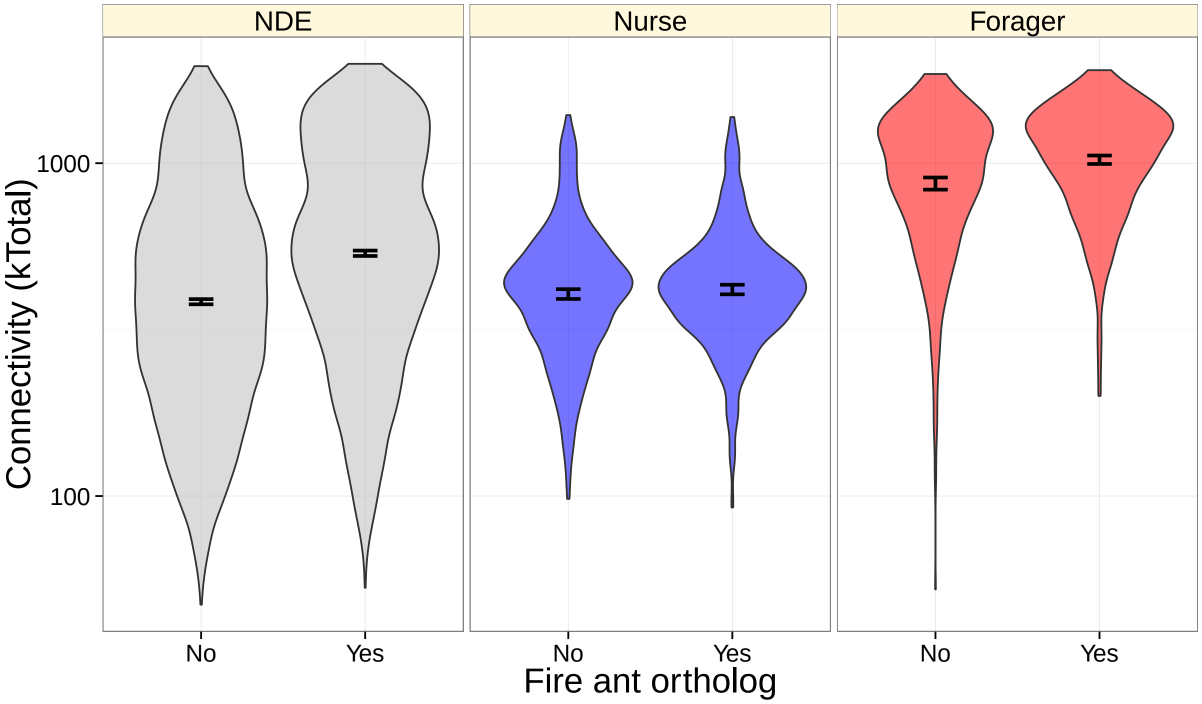


```
#expression vs. fire ant ortholog
antorthologexpression_plot<-ggplot(connectivity,aes(factor(homolog),
  fpkm,fill=task,alpha=0.2))+
  geom_violin()+facet_grid(.~task)+theme_bw()+
  ylab("Expression (FPKM)")+
  scale_y_log10(labels=fmt(),limits=c(1, 1e3))+
  xlab("Fire ant ortholog")+
  scale_fill_manual(values=c("grey","blue","red"))+
  theme(legend.position="none")+theme(plot.margin=unit(c(0,0.5,0.5,0.5), "cm"),
  axis.text.y = element_text(size=14),axis.text.x = element_text(size=14),
  axis.title.y = element_text(size=20),axis.title.x = element_text(size=20))+
  ggtitle("")+
  theme(strip.text.x = element_text(size=16),
        strip.background = element_rect(fill="cornsilk1"))+
  stat_summary(fun.data="mean_cl_boot", geom="errorbar",width=0.15,alpha=1,size=1,colour = "black")
antorthologexpression_plot
```

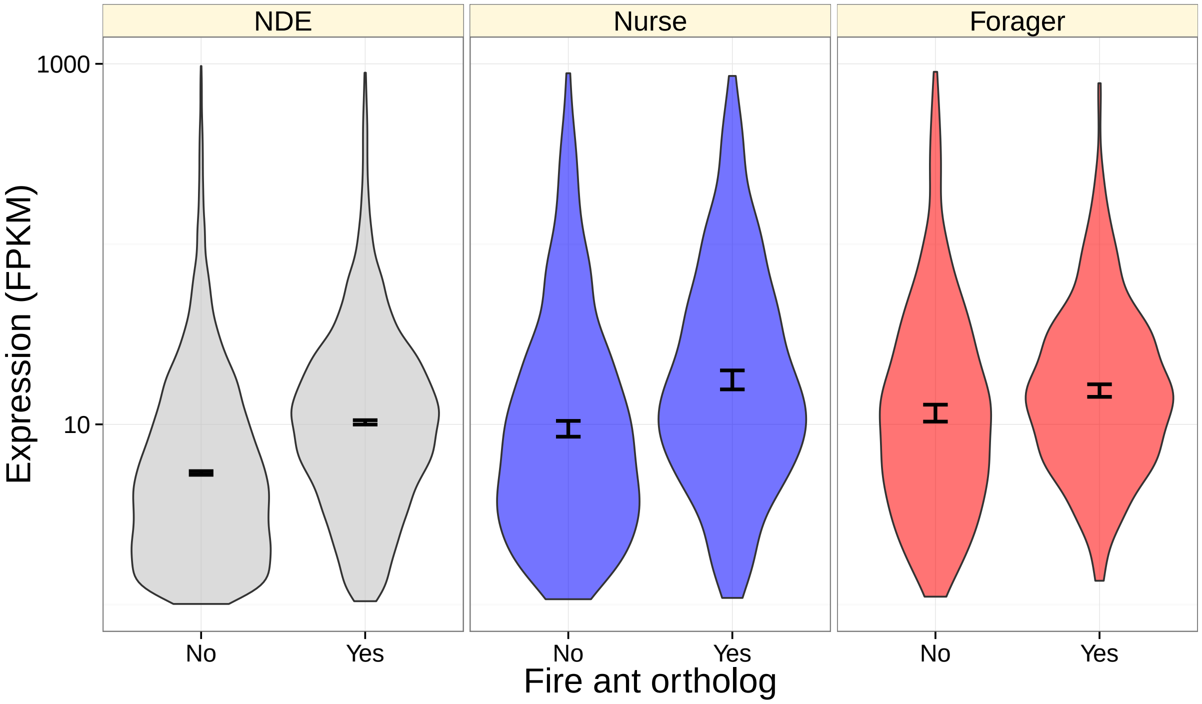


```
#expression vs. honey bee ortholog
beeorthologexpression_plot<-ggplot(connectivity,aes(factor(beehomolog),
  fpkm,fill=task,alpha=0.2))+
  geom_violin()+facet_grid(.~task)+theme_bw()+
  ylab("Expression (FPKM)")+
  scale_y_log10(labels=fmt(),limits=c(1, 1e3))+
  xlab("Honey bee ortholog")+
  scale_fill_manual(values=c("grey","blue","red"))+
  theme(legend.position="none")+theme(plot.margin=unit(c(0,0.5,0.5,0.5), "cm"),
  axis.text.y = element_text(size=14),axis.text.x = element_text(size=14),
  axis.title.y = element_text(size=20),axis.title.x = element_text(size=20))+
  ggtitle("")+
  theme(strip.text.x = element_text(size=16),
  strip.background = element_rect(fill="cornsilk1"))+
  stat_summary(fun.data="mean_cl_boot", geom="errorbar",width=0.15,alpha=1,size=1,colour = "black")
beeorthologexpression_plot
```

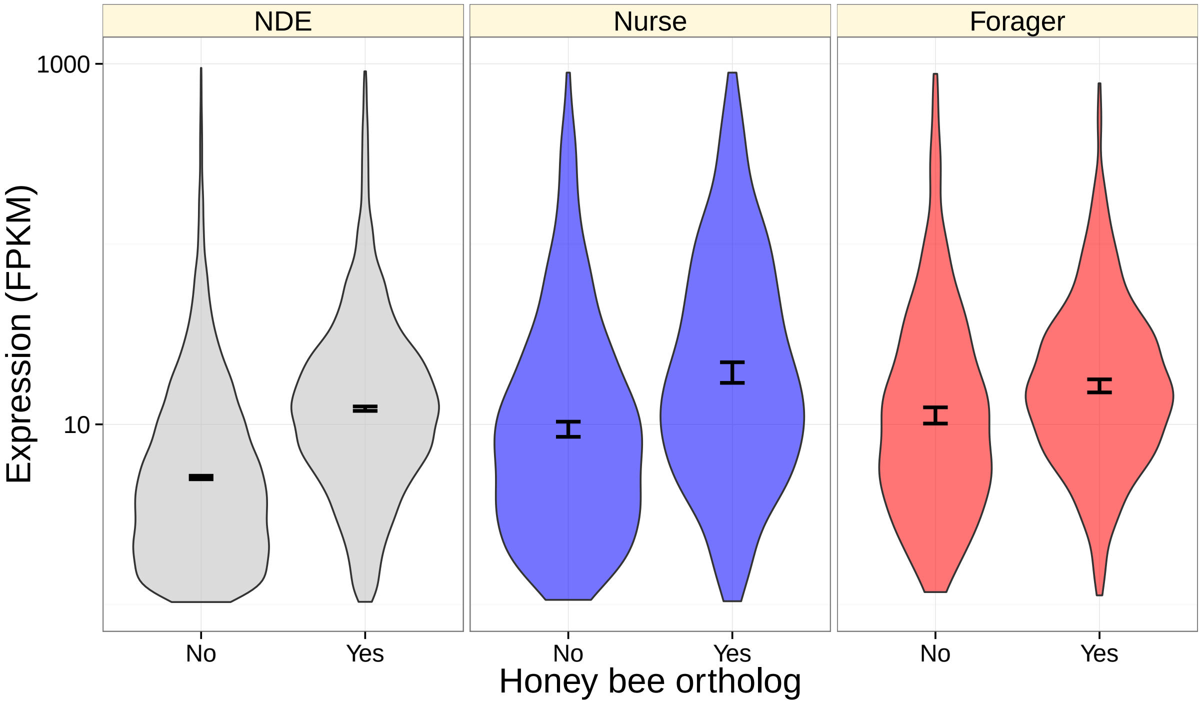


```r
#connectivity vs. behavioral category
connectivity_rate$task<-factor(connectivity_rate$task,labels=c("NDE","Nurse","Forager"))
connectivity_plot <- ggplot(connectivity_rate,aes(x=factor(task),y=kTotal,fill=task,alpha=0.2))+
  geom_violin()+theme_bw()+
  ylab("Connectivity (kTotal)")+scale_y_log10()+
  xlab("")+
  scale_fill_manual(values=c("grey","blue","red"))+
  theme(legend.position="none")+theme(plot.margin=unit(c(0,0.5,0,0), "cm"),
  axis.text.y = element_text(size=14),axis.text.x = element_text(size=14),
  axis.title.y = element_text(size=20),axis.title.x = element_text(size=20))+
  stat_summary(fun.data="mean_cl_boot", geom="errorbar",width=0.15,alpha=1,size=1,colour = "black")
connectivity_plot
```

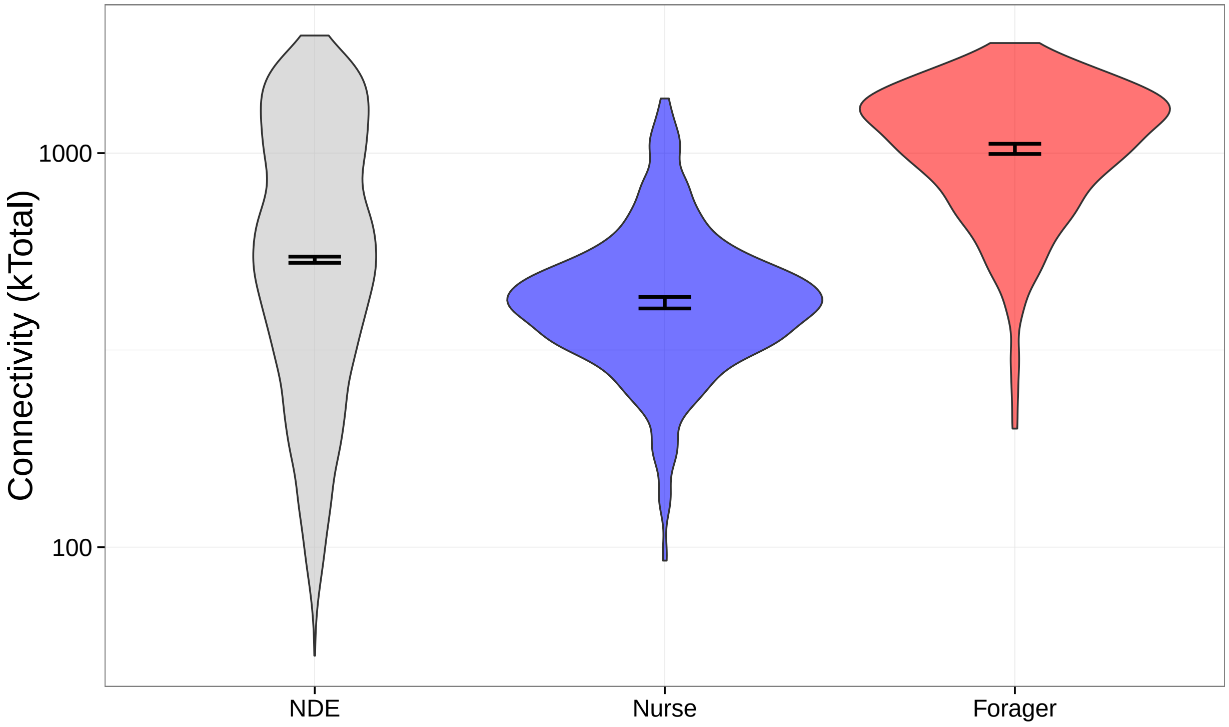


```r
#evolutionary rate vs. behavioral category
rate_plot<-ggplot(connectivity_rate,aes(x=factor(task),y=rate,fill=task,alpha=0.2))+
  geom_violin()+theme_bw()+
  ylab("Evolutionary rate (dN/dS)")+xlab("")+
  scale_y_log10(labels=fmt(),limits=c(5e-3, 3))+
  scale_fill_manual(values=c("grey","blue","red"))+
  theme(legend.position="none")+theme(plot.margin=unit(c(0,0.5,0,0), "cm"),
  axis.text.y = element_text(size=14),axis.text.x = element_text(size=14),
  axis.title.y = element_text(size=20),axis.title.x = element_text(size=20))+
  stat_summary(fun.data="mean_cl_boot", geom="errorbar"
  ,width=0.15,alpha=1,size=1,colour = "black")
rate_plot
```

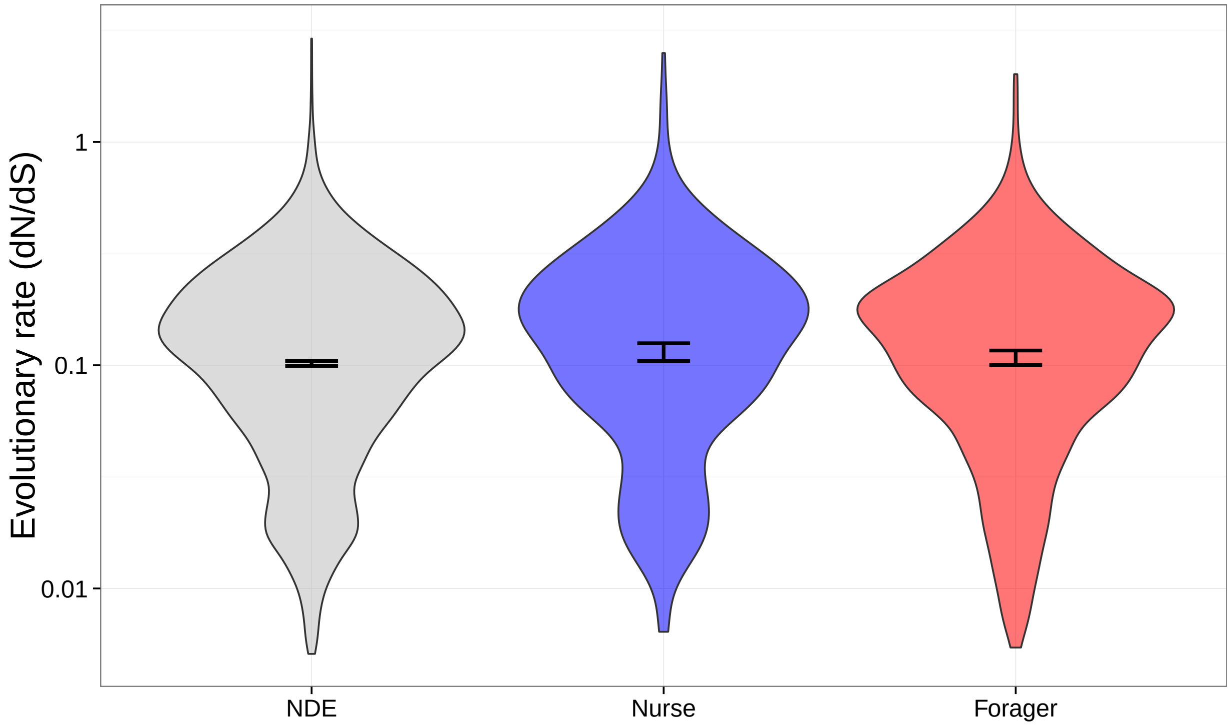


```
#expression vs. behavioral category
expression_plot<-ggplot(connectivity_rate,aes(x=factor(task),y=fpkm,fill=task,alpha=0.2))+
  geom_violin()+theme_bw()+
  ylab("Expression (FPKM)")+xlab("")+
  scale_y_log10(labels=fmt(),limits=c(1, 1e4))+
  scale_fill_manual(values=c("grey","blue","red"))+
  theme(legend.position="none")+theme(plot.margin=unit(c(0,0.5,0,0), "cm"),
  axis.text.y = element_text(size=14),axis.text.x = element_text(size=14),
  axis.title.y = element_text(size=20),axis.title.x = element_text(size=20))+
  stat_summary(fun.data="mean_cl_boot", geom="errorbar"
               ,width=0.15,alpha=1,size=1,colour = "black")
expression_plot
```

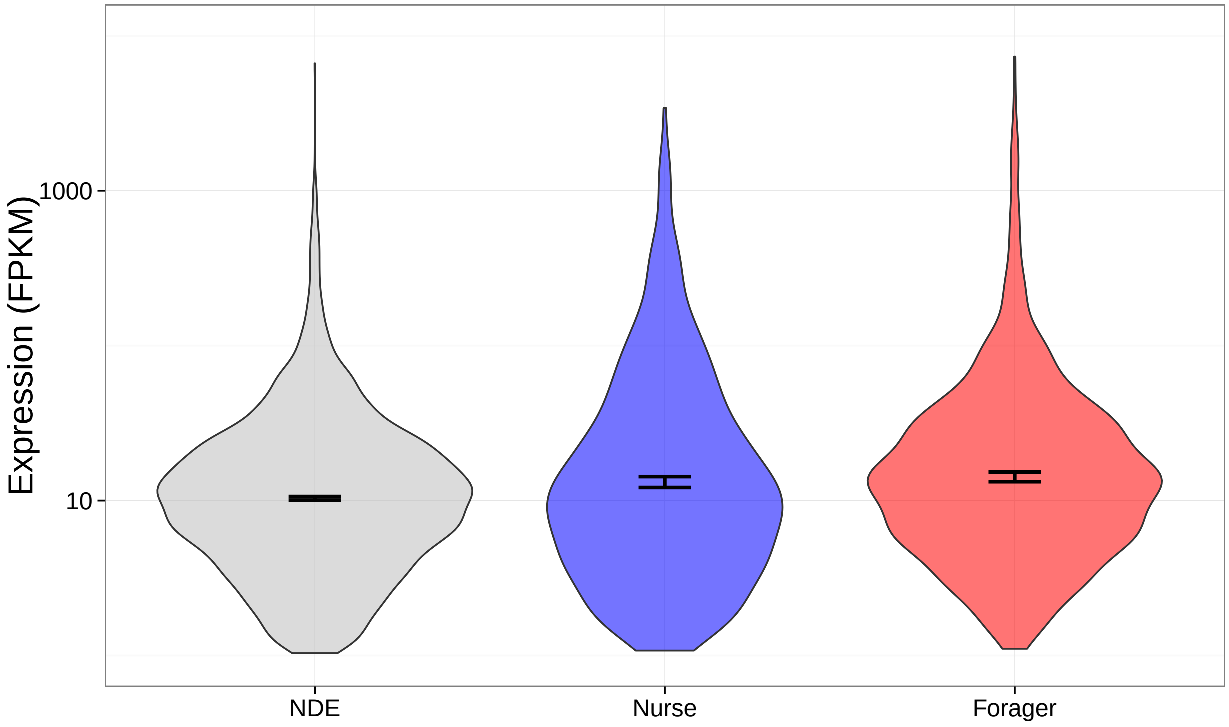


```
#fpkm vs connectivity
fpkm_connectivity_plot <- ggplot(connectivity_rate,aes(fpkm,kTotal,color=task))+
  geom_point(alpha=0.7)+scale_x_log10(limits=c(1, 1e4))+
  scale_y_log10(labels=fmt())+theme_bw()+
  ylab("Connectivity (kTotal)")+xlab("Expression (FPKM)")+
  stat_smooth(method="lm",se=FALSE,size=1.25,fullrange=TRUE,aes(group=task))+
  scale_color_manual(values=c("grey","blue","red"))+
  theme(legend.position="none")+theme(plot.margin=unit(c(0,0.5,0,0), "cm"),
  axis.text.y = element_text(size=14),axis.text.x = element_text(size=14),
  axis.title.y = element_text(size=20),axis.title.x = element_text(size=20))+
  ggtitle("")
fpkm_connectivity_plot
```

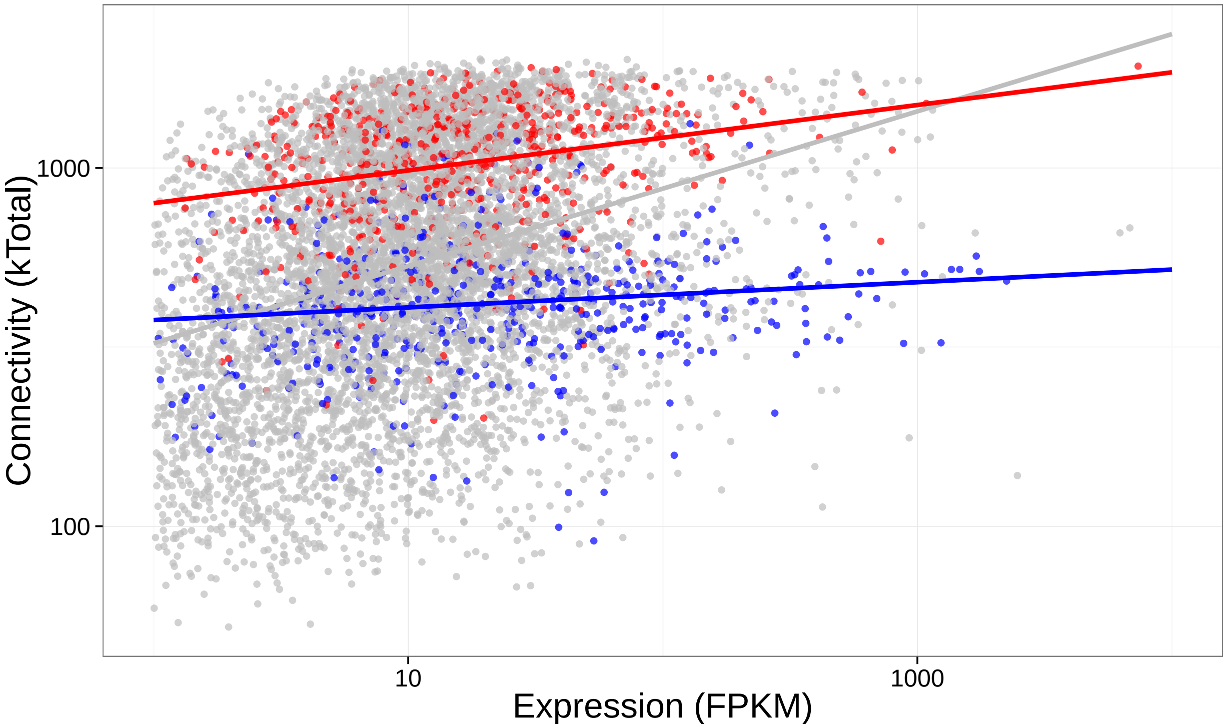


```
#rate and fpkm
rate_fpkm_plot <- ggplot(connectivity_rate,aes(fpkm,rate,color=task))+
  geom_point(alpha=0.7)+scale_x_log10(limits=c(1, 1e4))+
  scale_y_log10(labels=fmt(),limits=c(1e-3, 1))+theme_bw()+
  ylab("Evolutionary rate (dN/dS)")+xlab("Expression (FPKM)")+
  stat_smooth(method="lm",se=FALSE,size=1.25,fullrange=TRUE,aes(group=task))+
  scale_color_manual(values=c("grey","blue","red"))+
  theme(legend.position="none")+theme(plot.margin=unit(c(0,0.5,0,0), "cm"),
  axis.text.y = element_text(size=14),axis.text.x = element_text(size=14),
  axis.title.y = element_text(size=20),axis.title.x = element_text(size=20))+
  ggtitle("")
rate_fpkm_plot
```

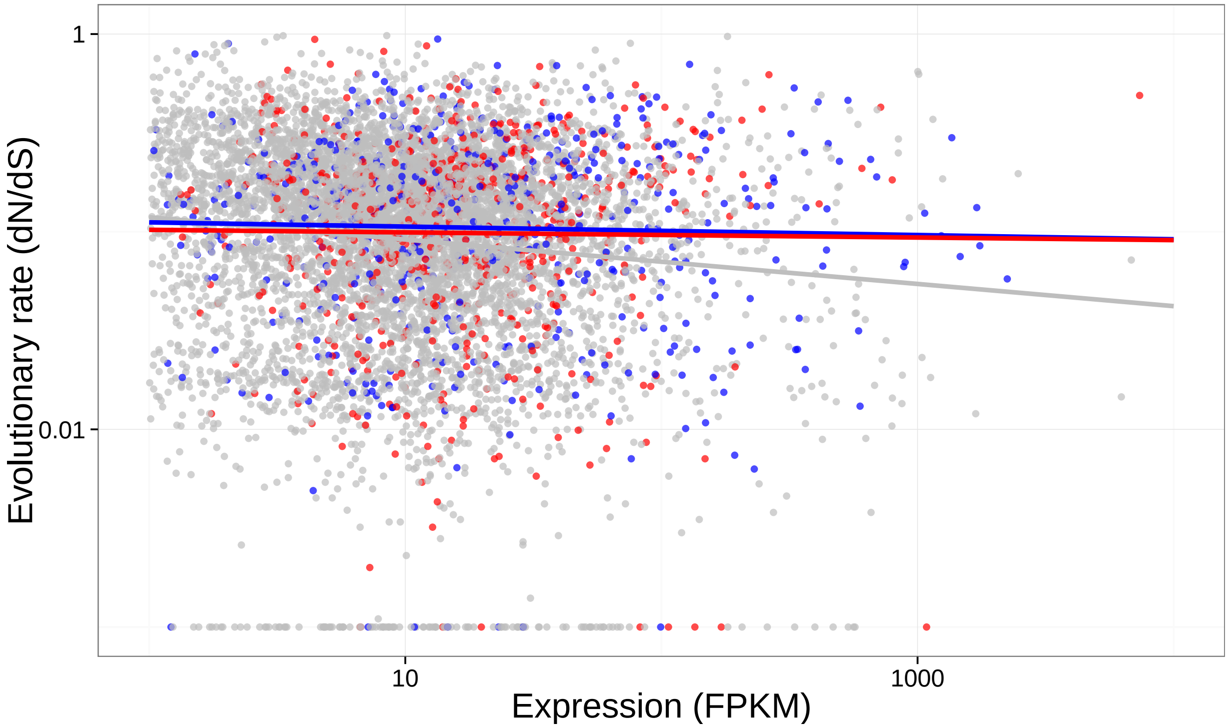


```
# evolutionary rates and connectivity
connectivity_rate_plot <- ggplot(connectivity_rate,aes(kTotal,rate,color=task))+
  geom_point(alpha=0.7)+scale_x_log10()+
  scale_y_log10(labels=fmt(),limits=c(1e-3, 1))+theme_bw()+
  ylab("Evolutionary rate (dN/dS)")+xlab("Connectivity (kTotal)")+
  stat_smooth(method="lm",se=FALSE,size=1.25,fullrange=TRUE,aes(group=task))+
  scale_color_manual(values=c("grey","blue","red"))+
  theme(legend.position="none")+theme(plot.margin=unit(c(0,0.5,0,0), "cm"),
  axis.text.y = element_text(size=14),axis.text.x = element_text(size=14),
  axis.title.y = element_text(size=20),axis.title.x = element_text(size=20))+
  ggtitle("")
connectivity_rate_plot
```

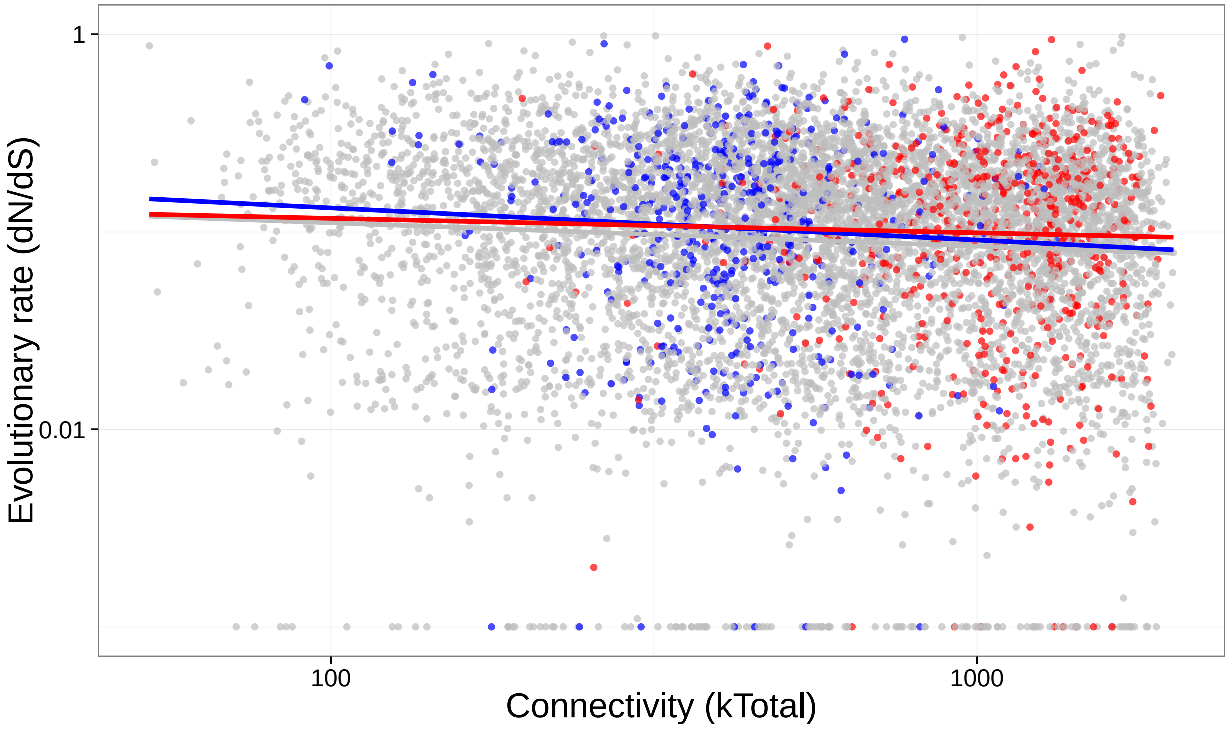

1
0.01
100
1000
Evolutionary rate (dN/dS)
Connectivity (kTotal)
